# Genomic evolution, recombination, and inter-strain diversity of chelonid alphaherpesvirus 5 from Florida and Hawaii green sea turtles with fibropapillomatosis

**DOI:** 10.7717/peerj.4386

**Published:** 2018-02-20

**Authors:** Cheryl L. Morrison, Luke Iwanowicz, Thierry M. Work, Elizabeth Fahsbender, Mya Breitbart, Cynthia Adams, Deb Iwanowicz, Lakyn Sanders, Mathias Ackermann, Robert S. Cornman

**Affiliations:** 1 National Fish Health Research Laboratory, Leetown Science Center, US Geological Survey, Kearneysville, WV, United States of America; 2 National Wildlife Health Center, Honolulu Field Station, US Geological Survey, Honolulu, HI, United States of America; 3 College of Marine Science, University of South Florida, St. Petersburg, FL, United States of America; 4 Institute of Virology, University of Zurich, Zurich, Switzerland; 5 Fort Collins Science Center, US Geological Survey, Fort Collins, CO, United States of America

**Keywords:** Green sea turtles, Fibropapillomatosis, Recombination, Protein divergence, Viral genomics, High-throughput sequencing, Phylogeography, Chelonid alphaherpesvirus 5

## Abstract

Chelonid alphaherpesvirus 5 (ChHV5) is a herpesvirus associated with fibropapillomatosis (FP) in sea turtles worldwide. Single-locus typing has previously shown differentiation between Atlantic and Pacific strains of this virus, with low variation within each geographic clade. However, a lack of multi-locus genomic sequence data hinders understanding of the rate and mechanisms of ChHV5 evolutionary divergence, as well as how these genomic changes may contribute to differences in disease manifestation. To assess genomic variation in ChHV5 among five Hawaii and three Florida green sea turtles, we used high-throughput short-read sequencing of long-range PCR products amplified from tumor tissue using primers designed from the single available ChHV5 reference genome from a Hawaii green sea turtle. This strategy recovered sequence data from both geographic regions for approximately 75% of the predicted ChHV5 coding sequences. The average nucleotide divergence between geographic populations was 1.5%; most of the substitutions were fixed differences between regions. Protein divergence was generally low (average 0.08%), and ranged between 0 and 5.3%. Several atypical genes originally identified and annotated in the reference genome were confirmed in ChHV5 genomes from both geographic locations. Unambiguous recombination events between geographic regions were identified, and clustering of private alleles suggests the prevalence of recombination in the evolutionary history of ChHV5. This study significantly increased the amount of sequence data available from ChHV5 strains, enabling informed selection of loci for future population genetic and natural history studies, and suggesting the (possibly latent) co-infection of individuals by well-differentiated geographic variants.

## Introduction

The neoplastic disease, fibropapillomatosis (FP) is present in sea turtles worldwide (Quackenbush et al., 1998; Lackovich et al., 1999), primarily affecting green sea turtles (*Chelonia mydas*; Smith & Coates, 1938) and to a lesser extent loggerhead (*Caretta caretta;* Harshbarger, 1991), olive ridley (*Lepidochelys olivacea;* Aguirre et al., 1999), Kemp’s ridley (*Lepidochelys kempii;* Barragan & Sarti, 1994), hawksbill (*Eretmochelys imbricate*; D’Amato & Moraes-Neto, 2000) and leatherback (*Dermochelys coriacea*; Huerta et al., 2002) sea turtles. FP manifests as external fibropapillomas or fibromas and internal fibromas, myxofibromas, or fibrosarcomas of low grade malignancy (Herbst, 1994) and when severe, can lead to immunosuppression (Work et al., 2001), secondary bacterial infections (Work et al., 2003), and death (Work et al., 2004). Fibropapillomatosis is now considered a panzootic in green turtles (Williams et al., 1994) where it is a leading cause of strandings in Hawaii (Aguirre et al., 1998; Chaloupka et al., 2008; Work et al., 2004) and Florida (Foley et al., 2005).

Transmission studies implicate an enveloped virus as the likely etiological agent of FP (Herbst et al., 1995) and herpesvirus-like particles are routinely observed via transmission electron microscopy in association with FP tumors (Jacobson et al., 1991). All available evidence suggests that chelonid alphaherpesvirus 5 (ChHV5) is the etiological agent of FP, with molecular studies showing a strong association between tumor presence and ChHV5 DNA (Lachovich et al., 1999; Lu et al., 2000; Quackenbush et al., 2001; Herbst et al., 2004; Ene et al., 2005; Duarte et al., 2012; Patrício et al., 2012). However, attempts to isolate ChHV5 in cell culture have been unsuccessful (Work et al., 2009), until recently when the virus was grown in organotypic skin cultures (Work et al., 2017). In cases such as this where the virus cannot easily be grown in culture, genomic sequencing presents a valuable strategy for investigating various aspects of viral ecology and evolution to supplement viral morphogenesis and other studies looking at pathogenesis of disease. Unfortunately, efforts to sequence the ChHV5 genome (Lu et al., 1999; Greenblatt et al., 2005b; Work et al., 2009) have been hindered by its latent lifestyle (Alfaro-Núňez et al., 2014), which maintains relatively low ChHV5 copy numbers compared to host DNA (Quackenbush et al., 2001) and precludes virion purification strategies to enrich for the viral genome prior to sequencing (Ng et al., 2009).

Obtaining sequence data from ChHV5 is an important goal because geographic differences between ChHV5 strains may be one potential factor in the differential gross manifestation (high prevalence of oral tumors) in Hawaiian green sea turtles (Aguirre et al., 2002) versus Florida green sea turtles (no oral tumors) (Foley et al., 2005), as well as the long-term decline of FP being observed in Hawaii but not Florida (Chaloupka, Balazs & Work, 2009). To date, molecular epizootiological studies of ChHV5 have focused on restricted and often non-overlapping suites of individual genes or small subsets of genes (Ene et al., 2005; Greenblatt et al., 2005a), resulting in a limited picture of the genetic variation among global populations of the virus. Nonetheless, such studies have shown that geographic structure instead of structuring exists among oceanographic regions, with ChHV5 phylogeographic patterns generally reflecting movements of the sea turtle host (Herbst et al., 2004; Ene et al., 2005; Patrício et al., 2012; Ariel et al., 2017), suggesting that ChHV5 has undergone region-specific co-evolution with sea turtle hosts (Jones et al., 2016).

In 2012, the first complete ChHV5 genome was sequenced by screening over 10,000 bacterial artificial chromosome (BAC) clones from the glottis tumor of a Hawaiian green sea turtle (Ackermann et al., 2012). Although the use of BAC clones is not efficient for sequencing large portions of the genome from numerous samples, the availability of this genome provides new avenues to explore the molecular epizootiology of FP that can overcome some of the methodological challenges described above. Here we utilized the available ChHV5 genome as a reference sequence to develop a simple long-range PCR (LR PCR) amplicon resequencing strategy based on the cost-effective Illumina MiSeq platform. Using this strategy, we sequenced partial viral genomes from the tumor tissue of multiple green sea turtles with FP from Florida (FL) and Hawaii (HI). This expanded collection of ChHV5 genomic sequence data, which is the largest presented to date, enabled the assessment of rates and patterns of change in a majority of ChHV5 protein coding genes as well as the opportunity to evaluate the concordance of multiple loci with existing hypotheses of biogeographical strain divergence derived from previous, more limited, datasets. Furthermore, characterization of the variability in particular viral protein coding genes sheds light on the evolutionary mechanisms that shape diversity and provides targets for future studies examining the linkages between geographic viral variants and differences in disease manifestation and dynamics.

## Materials & Methods

### DNA isolation, amplification and sequencing

Biopsies of skin tumors were obtained from green sea turtles that had stranded with fibropapillomatosis (Table 1). For HI samples, turtles found dead or humanely euthanized moribund individuals are classified as diagnostic specimens and are considered exempt according to the Animal Welfare Act. Therefore, the Chair of the US Geological Survey, National Wildlife Health Center Animal Care and Use Committee deemed it unnecessary to review or approve sampling. Florida tumor samples were collected according to Marine Turtle Permit MTP-16-233: Breitbart lab under protocol T-IS0001253 approved by the Institutional Animal Care and Use Committee, Research Integrity and Compliance, University of South Florida. A consent permit was issued from the Florida Fish and Wildlife Conservation Commission to transport marine turtle specimens out of the State.

**Table 1:** Green sea turtle (*Chelonia mydas*) tumor biopsy samples included in ChHV5 sequencing.

Case ID	Sample ID	Sample date	Location	Tumor location	Sampled by	Mapped^a^	Genome coverage^b^
21610	HI_21610	6∕15∕2011	Kaaawa, Honolulu, Oahu	Flipper	T. Work	92	42
21611	HI_21611	6∕20∕2011	Hookipa Beach, Maui	Flipper	T. Work	83	38
21533	HI_21533	2∕9∕2011	Hilo, Hawaii	Flipper	T. Work	78	82
12354	HI_12354	3∕25∕1996	Launiupoko Beach Park, Maui	Flipper	T. Work	90	72
12379	HI_12379	5∕24∕1996	Halananea, Maui	Flipper	T. Work	90	56
ALF20110705-01	FL_F5	9∕8∕2012	Indian River Co., FL	N/A	S. Hirama	92	81
PRB20120615-01, Uno	FL_G5	11∕8∕2012	Martin Co., FL	Flipper	R. Butts	92	69
7118, Gabriel	FL_USF	8∕16∕2007	Sarasota, FL	N/A	L. Byrd	19	68
Average						80	64

**Notes.**

a Percentage of filtered reads mapped to the reference genome.

b Percentage of genomic bases with threshold coverage (5×).

Sections of tumors were excised, placed in cryovials, and stored at −70 °C. DNA was extracted from approximately 10 mg of tumor tissue using DNeasy Blood and Tissue Kit (Qiagen, Valencia, CA, USA). DNA, quantified with the Qubit double-stranded (ds) DNA High Sensitivity (HS) Assay Kit (ThermoFisher Scientific, Grand Isle, NY, USA), were then normalized to 20–45 ng/µl, with one or both concentrations used for LR PCR.

The reference ChHV5 genome (sequenced by Ackermann et al., 2012; GenBank HQ870327) used for primer design in this study exhibited the typical gene order for a Class D genome of the *Scutavirus* genus within the subfamily *Alphaherpesvirinae*. Overall, this genome could be divided into unique long sequence (UL; 101,152 bp), unique short sequence (US; 13,319 bp) and inverted repeats that flanked the US (IRS; 8,831 bp each) (Thiry et al., 2005; Ackermann et al., 2012). In this study, LR PCR primers were designed from the ChHV5 reference genome using Geneious v 6.1.8 (Biomatters Ltd.) to amplify overlapping fragments of approximately 5 kb (kilobase pairs). Given difficulties in culturing and purifying the virus, using targeted ChHV5 LR PCR primers was important in order to avoid host DNA, which would overwhelm a shotgun sequencing approach, especially in the case of latent viruses present at low copy numbers. Primer sequences and their genomic coordinates are provided in Table S1.

Long-range PCRs were initially performed using the Qiagen LongRange PCR Kit (Qiagen, Valencia, CA) following the manufacturer protocol. Reactions containing 0.4 µM of each primer, 500 µM of dNTPs, 1U of LongRange PCR Enzyme Mix, 1X LongRange PCR Buffer, and 1 µl of 45 ng/ul DNA template in a 25 µl reaction were run on a Bio-Rad T100™ thermal cycler as follows: 93 °C for 3 min, followed by 35 cycles of (93 °C for 15 s, 62 °C for 30 s, and 68 °C for 5 min). For LR PCR primer pairs that failed to amplify in a few samples, the TaKaRa LA Taq polymerase kit (ClonTech, Palo Alto, CA), was used. Reactions containing 0.2 µM of each primer, 200 µM of dNTPs, 0.75 U of PrimeSTAR GXL DNA Polymerase, 1X PrimeSTAR GXL Buffer, and 2.5 µl of DNA template in a 25 µl reaction, were run on a Bio-Rad T100™ thermal cycler as follows: 93 °C for 3 min, followed by 30 cycles of (98 °C for 10 s, 60 °C for 10 s, and 68°for 5 min). PCRs that did not successfully amplify using the standard TaKaRa LA protocol were optimized according to the manufacturer’s troubleshooting suggestions, which included decreasing the template concentration from 2 ng/µl to 0.5 ng/µl, plus lengthening the last two phases of thermocycling to 60 °C for 15 s, and 68°for 9 min. LR PCR products were verified by gel electrophoresis. For products with multiple bands resulting from nonspecific priming, the band of the expected size was excised from the gel and purified using the UltraClean^®^ GelSpin^®^ DNA Extraction Kit (MoBio, Carlsbad, CA, USA). PCR products were quantified using the Qubit^®^ double-stranded (ds) DNA High Sensitivity (HS) Assay Kit (ThermoFisher Scientific, Grand Isle, NY, USA), then normalized to 1 ng/µl. Amplicons from each sample were then pooled in equal volumes, barcoded, and sequenced simultaneously. Multiple sequencing runs were performed as we explored different platforms and included additional samples (Table S2).

Two samples (FL_F5 and HI_21553) were run separately on an Ion Torrent Personal Genome Machine (PGM™; Life Technologies, Foster City, CA, USA). For each sample, 20 LR PCR products were analyzed separately with the Bioanalyzer 2100 (Agilent Technologies, Santa Clara, CA, USA) using the DNA1000 reagent kit. For each sample, PCR products were pooled in equimolar concentrations and frozen at −20 °C. Libraries were made from pooled PCR products for each sample following the manufacturer’s protocol for the Ion Xpress Plus Fragment Library Kit (Publication # 4471989, Revision N). Briefly, for both libraries, 50 ng of DNA was fragmented using the Ion Shear™ Plus reagents and purified with Agencourt^®^ AMPure Beads (Beckman Coulter, Indianapolis, IN, USA). Fragment sizes were determined using the Bioanalyzer 2100 (Agilent Technologies, Clara, CA, USA) with the HS DNA kit. The Ion Torrent adapters were ligated using DNA ligase, nick-translated and purified with Agencourt^®^ AMPure Beads (Beckman Coulter, Indianapolis, IN, USA). For the FL_F5 and HI_21553 libraries, 200 and 100 base read libraries, respectively, were size selected with the *E-gel*^®^ SizeSelect™ 2% gel system (http://www.invitrogen.com). Both libraries were quantified using quantitative PCR (qPCR) using the Ion Library TaqMan^®^ Quantitation Kit (Life Technologies). Within 24 h, the libraries were diluted and placed on the Ion OneTouch™ system (Life Technologies) for template preparation for the emulsion and enrichment of OT2 200 Ion Sphere™ particles (ISP) onto each library. The recovered template-positive Ion PGM template OT2 200 ISPs and library were cleaned with the OneTouch™ emulsion system (Life Technologies) following manufacturer protocols. Enrichment of the ISP was confirmed using the Qubit^®^ 2.0 fluorometric analysis and the IonSphere™ Quality Control Kit. The final libraries were prepared for sequencing using the Ion PGM™ Sequencing 200 Kit v2 (Publication no. MAN0007273) using the Ion 318 v2 and Ion 316 semiconductor chips for the FL and HI samples, respectively.

For Illumina MiSeq^®^ runs, cleaned PCR-products were quantified using the Qubit^®^ double-stranded (ds)DNA HS Assay Kit (ThermoFisher Scientific, Grand Isle, NY, USA) and normalized to 0.2 ng/µl using 10 mM Tris, pH 8.5. Libraries were prepared using 1 ng total input DNA and the Nextera^®^ XT DNA Library Prep Kit following the Illumina Library Preparation Guide (Illumina, San Diego, CA, USA). The only modification to the protocol was to the PCR clean-up step, in which we used 0.5x Agencourt^®^ AMPure XP beads (Beckman Coulter, Indianapolis, IN, USA) to select for a larger insert size. Final library size was validated using the BioAnalyzer HS DNA Kit (Agilent Technologies, Santa Clara, CA, USA). Prepared libraries were quantified using the Qubit^®^ dsDNA HS Assay Kit (ThermoFisher Scientific, Grand Isle, NY) and normalized to 4 nM using 10 mM Tris, pH 8.5. The pooled libraries were run at 15 pM with a 5% PhiX174 spike on the Illumina MiSeq^®^ with a 600 cycle v3 reagent kit (Illumina, San Diego, CA, USA).

### Data analysis

#### Initial bioinformatics

Machine-demultiplexed fastq files were imported into CLC Genomics Workbench v. 8 (https://www.qiagenbioinformatics.com) for trimming of exogenous sequences and low-quality base calls. Sequencing adapters were removed using the default scoring algorithm, bases were trimmed on an error probability of 0.05, and at most three ambiguous bases per read were allowed. Reads containing less than 50 bp after trimming were discarded. For Illumina paired-end reads, reads orphaned after the trimming step were discarded.

The combined filtered short-read data for each sample were mapped to the ChHV5 reference genome sequence (HQ878327). Mapping was performed with smalt (http://www.sanger.ac.uk/science/tools/smalt-0) version 0.7.6 with a word size of 13 and a step size of 5. Mappings for separate sequencing runs of the same biological sample were merged in samtools v. 1.3 (Li et al., 2009). Mappings were filtered to exclude those with Phred-scaled quality less than 20. Raw sequences were deposited in GenBank as a BioProject (ID: PRJNA360405), including biosample numbers SAMN06210144, SAMN06210145, SAMN06210146, SAMN06210147, SAMN06210148, SAMN06210149, SAMN06210150. For sample metadata, as well as processed alignment files associated with this project, see (Morrison et al., 2018).

Consensus sequences were generated from sam-formatted alignments using the samtools mpileup function and the view, call, and consensus functions of bcftools version 1.3.1 (https://samtools.github.io/bcftools). A consensus base was chosen at variable sites following the original samtools model (-c switch) rather than the multiallelic model, and IUPAC ambiguity codes were not used. Coverage was computed with the samtools depth function, and all sites in each sample with coverage less than 5× were converted to “N”. Open reading frames (ORFs) were parsed from consensus sequences based on the coordinates in Ackermann et al. (2012) with some minor corrections to ensure full representation of the final codons.

#### Polymorphism & divergence

For each ORF, sequences were confirmed by translating the nucleotide sequences into amino acid sequences in Geneious using the standard genetic code, then checking for stop codons within reading frames. Additionally, for each ORF, at least one nucleotide sequence was compared to data available in GenBank using the nucleotide BLAST search. Metrics of DNA polymorphism were computed using complete ORF sequences and the DNA Sequence Polymorphism software (DnaSP v5, Librado & Rozas, 2009) and MEGA7 (Kumar, Stecher & Tamura, 2016). The number of unique sequences, the proportion of segregating sites (Ps), and the nucleotide diversity (average number of nucleotide differences per site between two sequences (*π*; Nei, 1987)), were calculated for all sequences as well as for each regional set of sequences in MEGA7. DNA divergence was estimated in DnaSP by defining regional sequence sets and calculating (1) the average number of nucleotide substitutions per site (*D*_*XY*_, Nei, 1987, equation 10.20), and (2) numbers of synonymous (*Ks*) and nonsynonymous (*Ka*) substitutions per site (Nei & Gojobori, 1986, equations 1–3), using the universal genetic code. In order to find outlier clusters along the genome, the number of “private alleles” or “singletons”, i.e., consensus base calls that were unique to only one sample at a given nucleotide position, were extracted directly from the fasta-formatted consensus sequences in 500 bp genomic windows. In order to smooth the inherently stochastic processes involved in mutation, the count of private alleles per 500 bp window was Z-transformed on an approximately continuous distribution, allowing for outlier clusters of private alleles to be visualized.

#### Phylogenetic analyses

Each ORF was initially analyzed using the Geneious Tree Builder with Tamura–Nei (Tamura & Nei, 1993) genetic distances and the neighbor-joining tree building method (Saitou & Nei, 1987), and topologies were visually compared among individual ORF phylogenies. Since 11 of the 66 ORFs we sequenced were overlapping (HP14, HP15, HP19, F-UL26.5, HP22, HP23, HP24, HP25, HP26, HP31 and HP38), they were excluded from the alignment used to construct a global phylogenetic hypothesis. We then created a concatenated alignment of the remaining 55 unique ORFs to estimate a global phylogeny. Sequences with less than 50% of the ORF data present, plus those that were potentially recombinant (multiple sources of evidence were used as described below) were filtered from the alignment. Optimal partitioning schemes and nucleotide substitution models were selected following heuristic searches based upon gene and codon position in PartitionFinder (Lanfear et al., 2012). The optimal scheme was selected using the corrected akaike information criterion (AICc; Sullivan & Joyce, 2005).

Phylogenetic analyses of the manually curated, concatenated 55 ORF alignment (72,828 bp) were completed using MrBayes v3.2.6 x64 (Ronquist et al., 2012). First, the AICc favored partitioned dataset was run. Second, Bayesian model jumping was also employed in MrBayes, in which no model is selected, but instead sampling across all 203 possible general time-reversible rate matrices occurs according to their posterior probabilities (Ronquist et al., 2012). Each MrBayes analysis included five separate runs with four heated chains and 100,000 generations, sampling after every 500 generations, and diagnostics sampled every 1,000 generations and a burn-in fraction of 25%. Convergence among samples was monitored using the average standard deviation of split frequencies reported during the run, and by inspecting that the Potential Scale Reduction Factor (PSRF) diagnostic factor approached 1.0 (Ronquist et al., 2012). Phylogenetic trees were edited in FigTree v1.4.2 (Rambaut, 2014).

In order to compare the FL and HI ChHV5 variants sequenced in this study to strains previously documented in these geographic regions, sequence alignments were created for four gene regions previously utilized to determine strains: Amplicon IV and V, partial F-UL28 (glycoprotein B), and partial F-UL30 (DNApol; Herbst et al., 2004; Ene et al., 2005). The four alignments were concatenated into one alignment (6,160 bp in length) that contained 15 variants.

#### Selection & recombination

Tajima’s *D* statistic (Tajima, 1989) was calculated in DnaSP v5 (Librado & Rozas, 2009). Under neutral evolution (Kimura, 1983), the number of segregating sites and the average number of nucleotide differences have the same expected value; deviations from equality (a nonzero *D*) may reflect demography or natural selection (Tajima, 1989).

The relative proportion of nonsynonymous (*Ka*) versus synonymous substitutions (*Ks*) is a common measure of the rate at which protein-coding sequences evolve, providing an index of the average selective pressure on a coding region. Since most genes experience predominantly purifying selection over their evolutionary history, the *Ka*∕*Ks* ratio is usually less than one (equivalently, *Ka* − *Ks* < 0), whereas for coding sequences evolving neutrally this ratio should converge to 1 as samples become increasingly large (Makalowski & Boguski, 1998). A *Ka*∕*Ks* ratio significantly greater than one (equivalently, *Ka* − *Ks* > 0) reveals the accumulation of coding changes faster than expected under complete neutrality, implying positive selection. However, the latter test lacks power when the number of positively selected sites is small or occur only in certain lineages (Yang & Nielsen, 2002). The *Ka*∕*Ks* ratio is often estimated as the single parameter *ω* (omega) in a likelihood model fit to an explicit phylogenetic topology and mutational model (Yang et al., 2000). We used PAML 4.2 (Yang, 1997; Yang, 2007) to estimate *ω* for each ORF. We expect *ω* to be more sensitive than the *Ka*∕*Ks* ratio as it is fit to the phylogenetic topology estimated for the virus as a whole and includes an explicit mutational model. A single *ω* was estimated for each ORF, as we did not explore lineage- or site-specific models for this small number of samples.

For ORFs that had sequence data for all samples, we tested whether individual ORF phylogenies differed from the global phylogeny using the weighted non-parametric Shimodaira–Hasegawa test (WSH; Shimodaira, 1993; Shimodaira, 1998; Shimodaira & Hasagawa, 1999; Buckley et al., 2001), using the codeml program of the PAML 4.2 package (Yang, 1997; Yang, 2007).

Six tests of recombination (RDP, GENECONV, Bootscan, Maxchi, Chimaera, and 3Seq) were implemented in RDP4 (Martin et al., 2015) for genomic regions with high numbers of singletons. For each test, the ChHV5 reference genome (HQ878327) and the FLF5 variant were used to represent each of the two clades, with the exception of one test of a region of FLF5 as potentially recombinant, in which case the FLG5 sequence was chosen to represent the FL type sequence.

## Results

### Genomic re-sequencing using long-range PCR and high-throughput sequencing

**Figure 1:**
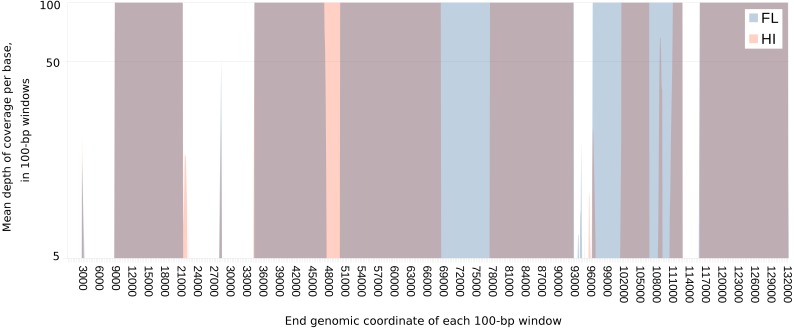
Extent of and geographic variation in the sequence recovery of the chelonid alphaherpesvirus 5 genome. For each tumor sample, the read coverage at each genomic site of the virus was extracted with the mpileup function of samtools (Li et al., 2009). The mean coverage in 100-bp windows was then determined for each sample by summing these coverage values and dividing by 100. The figure overlays the median of the means for samples from each region (Florida or Hawaii), plotted on a log scale. Several genomic regions were recovered only from Florida (blue) or Hawaii (orange). Genomic regions that are recovered from both FL and HI samples appear purple. The minimum value of the vertical axis is set at five merely to emphasize that individual sites with coverage less than this value were masked in each sample. The maximum value of the vertical axis (100×) was chosen to illustrate the range of variation in coverage depth while demonstrating that most sites have abundant coverage. The actual maximum coverage for any given sample was capped by default at 8,000× by the mpileup function. As a result, the figure does not indicate the large variation in absolute coverage attributable to amplification efficiency.

The amplification strategy was applied to three tumor samples from FL and five from HI (Table 1). Thirty-three of 40 LR PCR primer sets (82.5%) amplified a product of the expected length from at least one sample (Table S2), resulting in the resequencing of ∼73 and 57% of the FL and HI genomes respectively. Variable recovery of amplification products can be seen in the uneven depth of coverage across the genome (Fig. 1), such that not all ORFs were completely recovered for all samples. Regardless, over 75% of predicted protein coding genes were recovered from at least two samples: 7 of 11 ORFs in the US region, and 59 of 76 ORFs from the UL region of the alphaherpesvirus genome (Fig. 1, Table 2, see Morrison et al., 2018 for metadata and sequence alignments). At least two unique consensus sequences were found for every gene, with a maximum of five (F-US1, F-US3A, HP37 and HP38) and an average of 3.2 distinct sequences per gene (Table 2, Unique). For some primer pairs, (e.g., ChHV5-LSC-02 and 03, HI/FL, Table S1), we saw consistent geographic differences in primer success, suggesting sequence or structural polymorphisms between the two clades that were not ascertainable using this strategy.

**Table 2:** ChHV5 sequence diversity statistics for putative protein-coding genes from green sea turtles with fibropapillomatosis sampled in Florida (FL) and Hawaii (HI).

Designation^a^	GenBank^a^	Predicted features^a^	Complete sequences	*n*^b^	Unique^c^	*P*s^d^	*π*^e^	*π*^e^ (FL)	*π*^e^ (HI)	Tajima’s *D*^f^
F-US2	AHA93306	Ser/Arg-rich protein (pr)	6HI/3FL	891	3	0.116	0.029	0.071	0.000	−1.708
F-US1	AHA93307	Secretory pathway	6HI/3FL	2,008*	5	0.098	0.023	0.062	0.000	2.149
F-US8	AHA93301	Glycoprotein E	6HI/3FL	1,623	2	0.014	0.007	0.000	0.000	1.566
HP10	AHA93302	Nuclear localization	6HI/3FL	468	2	0.011	0.005	0.000	0.000	1.52
F-US4	AHA93303	Glycoprotein D	6HI/3FL	798	3	0.023	0.010	0.009	0.000	0.729
F-US3B	AHA93304	Cdk2 cyclin-dep kinase 2	6HI/3FL	942	3	0.058	0.015	0.034	0.000	−1.491
F-US3A	AHA93305	US3 protein kinase protein	6HI/3FL	1,152	5	0.056	0.015	0.031	0.000	−1.435
F-UL01	AHA93319	Glycoprotein L	4HI/3FL	380	2	0.008	0.004	0.000	0.000	1.811
F-UL02	AHA93320	Uracil DNA glycosylase	4HI/3FL	842	2	0.015	0.009	0.000	0.000	2.191
F-UL03	AHA93321	Nuclear phosphoprotein	4HI/3FL	686	3	0.007	0.004	0.001	0.000	1.288
F-UL04	AHA93322	Nuclear protein	4HI/3FL	590	3	0.003	0.001	0.001	0.000	0.206
F-UL05	AHA93323	DNA helicase/primase	4HI/3FL	2,538	3	0.011	0.006	0.001	0.000	2.176
HP14	AHA93324	Hypothetical Protein (HP)	4HI/3FL	624	2	0.010	0.005	0.000	0.000	2.032
F-UL06	AHA93325	Capsid portal pr	4HI/3FL	1,986	2	0.005	0.003	0.000	0.000	2.146
HP15	AHA93326	HP nuclear	4HI/3FL	515	2	0.004	0.002	0.000	0.000	1.645
F-UL07	AHA93327	Herpes UL7 superfamily	4HI/3FL	915	3	0.061	0.021	0.033	0.000	−0.942
F-UL08	AHA93328	Helicase-primase	4HI/3FL	2,244	4	0.050	0.018	0.025	0.000	−0.719
F-UL09	AHA93329	Origin binding pr	4HI/1FL	2,477	2	0.015	0.006	0.000	0.000	−1.377
F-UL10	AHA93330	Glycoprotein M	4HI/1FL	1,265	2	0.004	0.002	0.000	0.000	−2.023
F-UL11	AHA93331	Myristylated pr	4HI/1FL	284	2	0.007	0.003	0.000	0.000	−0.973
F-UL12	AHA93332	YqaJ-like recombinase	4HI/1FL	1,679	3	0.005	0.002	0.000	0.000	−0.987
F-UL17	AHA93337	DNA packaging tegument pr	5HI/3FL	1,956	3	0.037	0.019	0.000	0.006	1.085
F-UL15B	AHA93338	DNA packing pr	6HI/3FL	1,071	2	0.029	0.014	0.000	0.000	1.823
HP17	AHA93339	HP	6HI/3FL	468	2	0.064	0.032	0.000	0.000	1.8
HP18	AHA93340	HP, RLSA-like (human)	6HI/3FL	696	3	0.011	0.006	0.001	0.000	1.458
HP19	AHA93341	HP, RL6-like (human)	6HI/3FL	483	2	0.025	0.012	0.000	0.000	1.706
HP20	AHA93342	HP, virion transactivator	6HI/3FL	501	2	0.010	0.005	0.000	0.000	1.520
F-UL18	AHA93343	Viral capsid pr VP23	6HI/3FL	992	3	0.034	0.016	0.003	0.000	1.518
F-UL19	AHA93344	Major capsid pr	6HI/3FL	4,152	3	0.015	0.007	0.000	0.000	1.689
F-UL20	AHA93345	Egress pr UL20	6HI/3FL	630	2	0.008	0.004	0.000	0.000	1.520
F-UL21	AHA93346	Tegument pr	6HI/3FL	1,403	4	0.014	0.006	0.003	0.000	1.058
F-UL26	AHA93352	Capsid maturation protease	2HI/2FL	1,650	3	0.012	0.007	0.003	0.000	1.452
F-UL26.5	AHA93353	Virion scaffolding pr	2HI/2FL	882	3	0.014	0.009	0.002	0.000	1.727
F-UL27	AHA93356	Glycoprotein B	2HI/2FL	2,565	2	0.010	0.007	0.000	0.000	2.291
HP22	AHA93354	HP	2HI/2FL	687	2	0.010	0.007	0.000	0.000	2.180
HP23	AHA93355	C3 precursor pr	2HI/3FL	870	3	0.013	0.008	0.001	0.000	−1.231
F-UL28	AHA93357	DNA cleavage/packaging pr	4HI/3FL	2,253	2	0.015	0.008	0.000	0.000	−1.704
F-UL29	AHA93358	Single-stranded binding pr	4HI/3FL	3,588	2	0.012	0.007	0.000	0.000	−1.139
HP24	AHA93359	HP 6-phosphofructokinase	4HI/3FL	942	2	0.006	0.004	0.000	0.000	1.685
F-UL30	AHA93360	DNA polymerase subunit	3HI/3FL	3,453	3	0.020	0.012	0.000	0.000	1.78
F-UL31	AHA93361	UL31 Nuclear egress lamina pr	4HI/3FL	924	3	0.018	0.010	0.001	0.000	2.151
F-UL33	AHA93362	DNA cleavage/packaging pr	4HI/3FL	336	2	0.003	0.002	0.000	0.000	1.342
F-UL34	AHA93363	Membrane phosphoprotein	4HI/3FL	795	2	0.013	0.007	0.000	0.000	2.146
F-UL35	AHA93364	VP26 capsid pr	4HI/2FL	366	2	0.008	0.004	0.000	0.000	−0.302
F-UL38	AHA93367	Capsid shell pr VP19C	4HI/3FL	1,326	3	0.013	0.007	0.001	0.000	−1.001
HP25	AHA93368	HP bipartite NLS	4HI/3FL	510	2	0.010	0.006	0.000	0.000	−1.042
F-UL41	AHA93369	Tegument host shutoff pr	4HI/3FL	1,179	4	0.014	0.008	0.001	0.000	−1.121
F-UL42	AHA93370	DNA polymerase processivity	2HI/3FL	1,076	3	0.008	0.005	0.001	0.000	−1.535
F-UL43	AHA93371	Gallid UL43-like pr	2HI/3FL	1,226	3	0.015	0.009	0.001	0.000	−0.617
HP26	AHA93372	HP	2HI/3FL	491	2	0.010	0.006	0.000	0.000	−0.779
HP27	AHA93373	HP protein IG	2HI/3FL	653	2	0.012	0.007	0.000	0.000	−1.155
F-UL53	AHA93374	Glycoprotein K	2HI/3FL	1,061	3	0.012	0.007	0.000	0.001	−1.12
F-UL52	AEZ68791	UL52 helicase-primase subunit	6HI/3FL	1,372*	3	0.022	0.011	0.000	0.000	−1.346
HP30	AHA93377	HP	6HI/3FL	536	2	0.011	0.006	0.000	0.000	1.566
F-lec1	AEZ68794	C-type lectin	6HI/3FL	626	2	0.009	0.005	0.000	0.000	1.52
F-lec2	AEZ68793	C-type lectin domain family	6HI/3FL	530	2	0.013	0.007	0.000	0.000	1.601
F-sial	ABX60166	Glycosyltransferase family 29	6HI/3FL	963	2	0.012	0.006	0.000	0.000	1.701
F-Nec2	AHA93379	Like CD155 and CD112	6HI/3FL	1,740	2	0.010	0.005	0.000	0.000	1.747
HP31	AHA93378	HP Tau-tubulin kinase 1	6HI/3FL	525	2	0.011	0.006	0.000	0.000	1.566
HP32	AHA93380	HP Immunoglobulin V-set	6HI/3FL	1,686	3	0.011	0.005	0.000	0.000	1.549
HP33	AHA93381	HP	6HI/3FL	462	2	0.002	0.001	0.000	0.000	0.986
HP34	AHA93382	HP	4HI/3FL	474	2	0.006	0.004	0.000	0.000	1.645
HP35	AHA93383	HP heptad repeat regions	4HI/3FL	1,440	3	0.019	0.011	0.000	0.042	0.321
HP36	AHA93384	HP protease	5HI/3FL	453	2	0.004	0.003	0.000	0.000	1.65
HP37	AHA93385	HP ICP4 DNA- RNA- binding	4HI/2FL	2,094	5	0.025	0.011	0.001	0.001	0.289
HP38	AHA93386	HP ICP4	4HI/2FL	744	5	0.048	0.025	0.001	0.001	1.256
Total/Average				74,357	2.6	0.019	0.009	0.004	0.001	

**Notes.**

a Predicted features following Ackermann et al. (2012).

b *n*, number of sites.

c Unique, number of unique sequences.

d *P*s, Proportion of segregating sites, or the number of segregating sites/n.

e *π*, average number of substitutions per site (nucleotide diversity).

f Tajima’s D (Tajima, 1989) test statistic for the relationship between the number of segregating sites and the average number of nucleotide differences, expected to be correlated under neutral evolution.

### Genome-wide single nucleotide polymorphisms confirm broad divergence of Atlantic and Pacific strains

Most sequence variation was between regional ChHV5 strains rather than within a location, as expected based on previous phylogenetic analyses (Herbst et al., 2004; Greenblatt et al., 2005a; Greenblatt et al., 2005b; Ene et al., 2005; Patrício et al., 2012; Rodenbusch et al., 2012; Alfaro-Núňez et al., 2014). A total of 1,001 fixed differences was observed between Hawaiian and Floridian samples across the 66 sequenced proteins. All variable sites in over half of examined genes (35/66) were fixed differences between the two geographic strains (Table 3: Fixed), while few variable sites were detected within strains (Table 2: *π* FL, *π* HI). ORFs that exhibited the highest number of fixed differences included those of a DNA packing protein (F-UL17), a major capsid protein (F-UL19), a single-stranded binding protein (F-UL29), DNA polymerase (F-UL30), and a hypothetical protein (HP) with DNA- and RNA- binding domains (HP37; Table 3; Fixed).

**Table 3:** ChHV5 sequence divergence statistics for putative protein-coding genes from green sea turtles with fibropapillomatosis sampled in Florida (FL) and Hawaii (HI).

Designation^a^	Seqs	Fixed^b^	*Dxy* (JC)^c^	Codons	*Ks*^d^	*Ka*^e^	*Ka*∕*Ks*	*ω*^f^	WSH^g^	Mask^h^
F-US2	9	9	0.049	297	0.107	0.025	0.223	0.263	−1	FLUSF
F-US1	4	13	0.006	700	0.012	0.004	0.371	0.432	−1	FLUSF
F-US8	9	22	0.014	540	0.0362	0.007	0.176	0.181	−1	none
HP10	9	5	0.011	155	0.027	0.006	0.212	0.207	−1	none
F-US4	9	7	0.018	265	0.037	0.011	0.303	0.413	−1	FLF5
F-US3B	9	7	0.026	313	0.091	0.004	0.038	0.051	0.309	FLUSF
F-US3A	9	10	0.025	383	0.072	0.009	0.125	0.101	−1	FLUSF
F-UL01	7	3	0.008	126	0.033	0	0	0.000		none
F-UL02	7	13	0.016	281	0.047	0.005	0.098	0.116		none
F-UL03	7	4	0.006	228	0.005	0.007	1.254	1.222		none
F-UL04	7	1	0.002	196	0.009	0	0	0.000		none
F-UL05	7	25	0.010	845	0.030	0.004	0.116	0.090		none
HP14	7	6	0.010	207	0.006	0.011	1.728	2.559		none
F-UL06	7	10	0.005	661	0.010	0.003	0.352	0.308		none
HP15	7	2	0.004	171	0.007	0.003	0.402	0.697		none
F-UL07	7	11	0.029	304	0.079	0.012	0.139	0.152		FLUSF
F-UL08	7	27	0.026	747	0.056	0.014	0.24	0.254		FLUSF
F-UL09	5	37	0.015	825	0.043	0.005	0.111	0.112		FLUSF
F-UL10	5	5	0.004	421	0.013	0.001	0.085	0.087		none
F-UL11	5	2	0.007	94	0.014	0.005	0.348	0.344		none
F-UL12	5	8	0.005	559	0.012	0.003	0.219	0.246		HI21610
F-UL17	8	41	0.033	651	0.076	0.017	0.216	0.273	−1	HI21610
F-UL15B	9	31	0.030	356	0.097	0.006	0.06	0.071	−1	none
HP17	9	30	0.067	155	0.142	0.037	0.24	0.262	−1	none
HP18	9	7	0.011	231	0.022	0.007	0.324	0.423	−1	none
HP19	9	12	0.025	160	0.060	0.014	0.227	0.241		none
HP20	9	5	0.010	166	0.027	0.005	0.19	0.232	−1	none
F-UL18	9	29	0.033	330	0.110	0.005	0.042	0.054	−1	none
F-UL19	9	59	0.015	1,383	0.052	0.002	0.036	0.034	−1	none
F-UL20	9	5	0.008	209	0.031	0	0	0.000	−1	none
F-UL21	9	13	0.012	467	0.029	0.006	0.185	0.239	−1	none
F-UL26	4	14	0.010	549	0.018	0.007	0.404	0.359		none
F-UL26.5	4	10	0.013	293	0.019	0.010	0.523	0.578		none
F-UL27	4	26	0.010	852	0.031	0.004	0.114	0.122		none
HP22	4	6	0.009	229	0.006	0.010	1.734	2.570		none
HP23	5	10	0.012	289	0	0.017	na	999		HI12354
F-UL28	7	33	0.015	750	0.034	0.008	0.244	0.317	0.08	HI12354, −79*
F-UL29	7	43	0.012	1,195	0.044	0.002	0.033	0.032	0.075	HI12354, −79*
HP24	7	6	0.006	313	0.012	0.004	0.343	0.486		HI12354
F-UL30	5	67	0.020	1,151	0.052	0.009	0.164	0.182	0.142	HI12354, −79*
F-UL31	7	16	0.018	307	0.059	0.004	0.072	0.078		HI21610*
F-UL33	7	1	0.003	111	0.012	0	0	0.000		none
F-UL34	7	10	0.013	264	0.038	0.004	0.11	0.090		HI21611*
F-UL35	6	3	0.008	121	0.021	0.004	0.176	0.247		none
F-UL38	7	16	0.012	441	0.042	0.002	0.047	0.049		HI12354
HP25	8	0	0.012	150	0.010	0.013	1.343	0.855		HI12354
F-UL41	7	15	0.014	392	0.041	0.005	0.114	0.177		HI12354
F-UL42	5	8	0.008	358	0.018	0.004	0.222	0.258		HI12354
F-UL43	5	18	0.015	408	0.028	0.010	0.35	0.356		HI12354, −79*
HP26	6	0	0.017	163	0.015	0.018	1.205	0.923		HI12354
HP27	5	8	0.012	217	0.031	0.006	0.193	0.221		HI12354, −79*
F-UL53	5	12	0.012	353	0.038	0.003	0.066	0.067		HI12354
F-UL52	9	29	0.022	458	0.070	0.004	0.058	0.091	0	HI12354, −79
HP30	9	6	0.011	178	0.027	0.006	0.226	0.146	−1	none
F-lec1	9	5	0.009	178	0.016	0.007	0.475	0.560	−1	none
F-lec2	9	7	0.013	176	0.016	0.012	0.747	1.064	−1	none
F-sial	9	12	0.013	320	0.030	0.006	0.179	0.246	−1	none
F-Nec2	9	17	0.010	579	0.025	0.005	0.208	0.185	−1	none
HP31	9	6	0.012	173	0.016	0.010	0.638	1.131		none
HP32	9	17	0.010	561	0.025	0.005	0.201	0.209	−1	none
HP33	9	1	0.002	153	0.009	0	0	0.000	−1	none
HP34	7	3	0.006	157	0.009	0.006	0.628	0.910		none
HP35	7	27	0.019	479	0.049	0.009	0.184	0.213		HI21610*
HP36	7	2	0.004	150	0.016	0	0	0.000		none
HP37	6	48	0.025	697	0.0411	0.0186	0.446	0.434		none
HP38	6	34	0.048	246	0.0329	0.053	1.619	1.820		FLUSF*
Avg/Total*	7.17	15.08	0.015	25,837*	0.00437	0.00080				

**Notes.**

a Predicted features following Ackermann et al. (2012); see Table 2 for additional information.

b Fixed, number of sites at which all of the sequences in one population are different from all of the sequences in the second population ( Hey, 1991).

c *Dxy*(JC), average proportion of nucleotide differences between populations or species with Jukes & Cantor correction (Nei, 1987).

d *Ks*, the number of synonymous substitutions per synonymous site (Nei & Gojobori, 1986).

e *Ka* the number of nonsynonymous substitutions per nonsynonymous site (Nei & Gojobori, 1986).

f *ω Ka*∕*Ks* fit to an explicit phylogenetic topology in a likelihood framework (Yang et al., 2000).

g WSH, weighted Shimodaira/Hasegawa (Shimodaira, 1993; Shimodaira, 1998; Shimodaira & Hasagawa, 1999; Buckley et al., 2001) non-parametric test of whether individual phylogenies were significantly different relative to the global phylogeny.

h Mask indicates that the sequence was masked in calculations of the above statistics and in the global phylogenetic hypothesis due to possible cases of recombination or *missing ≥50% of data.

**Figure 2:**
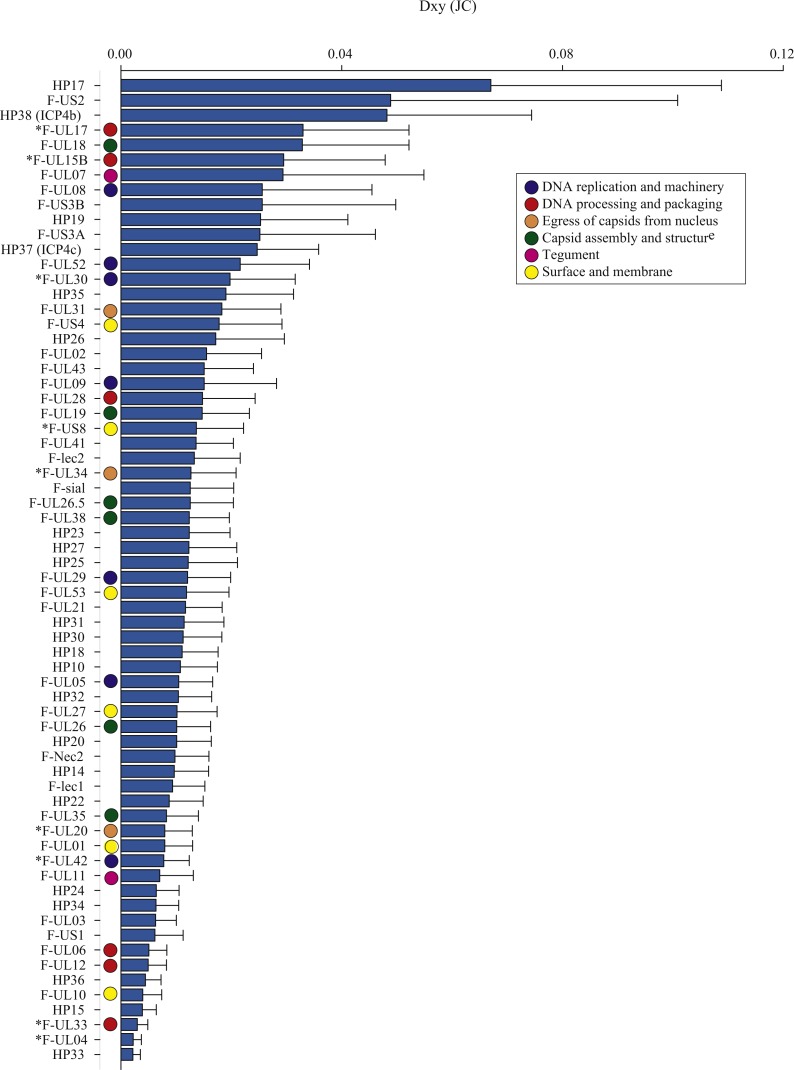
The average number of nucleotide substitutions per site (*Dxy*) between Florida and Hawaii geographic strains of ChHV5.

The average number of nucleotide differences per site between geographic groups (*Dxy*; Nei, 1987) varied across ORFs, from 0.002 (F-UL04 nuclear protein and HP33) to 0.067 (HP17), averaging 0.015 (Table 3, *Dxy*; Fig. 2). Some proteins had notably greater levels of geographic differentiation than average. For example, proteins with inter-region *Dxy* were more than twice the average including the Ser/Arg-rich protein F-US2, the viral capsid proteins F-UL17 and F-UL18, plus two HPs (HP17 and HP38, Table 3, *Dxy*, range 3.3–6.7%; Fig. 2).

### Divergence by protein function

Protein divergence rates can indicate, for example, the severity of functional constraint, and the occurrence of diversifying selection in different environments. We evaluated protein divergence in terms of the estimated nonsynonymous substitution rate (*Ka*) as well as the relative rate of nonsynonymous to synonymous substitutions (*ω*, Yang et al., 2000). Relative rates of nonsynonymous substitution less than, equal to, or greater than 1 indicate that proteins are evolving under purifying selection, neutrality, or positive selection respectively (averaged across sites and branches; Yang et al., 2000). As expected, these two metrics were highly correlated (Pearson’s *r* = 0.948; see Table 3 *Ka*∕*Ks* and *ω*).

Estimates of protein divergence ranged from 0 (F-UL01, F-UL04, F-UL20, F-UL33 HP33, HP36) to 5.3% (HP38) in the 66 ChHV5 protein-coding genes examined. Several of the most conserved genes are involved with core functions, such as DNA replication, DNA processing and packaging, surface and membrane proteins, and capsid assembly and structure (see McGeoch, Rixon & Davison, 2006). For example, several core proteins were nearly identical at the amino acid level between Floridian and Hawaiian strains, including glycoprotein L (F-UL01), a gene involved in DNA cleavage/packaging (F-UL33), a nuclear protein (F-UL04), an egress protein (F-UL20; Table 3, *Ka*), along with two hypothetical proteins with unknown function (HP33 and HP36). Given the conserved nature of HP33, HP36 and F-UL04, further investigation of their biological role is warranted. Many of the genes that appear conserved among ChHV5 variants, such as the DNA cleavage/packaging protein (F-UL33), glycoprotein L (F-UL01), and the F-UL20 egress protein (Table 3, *Ka*), are also highly conserved among strains of the alphaherpesvirus HHV-1 (Szpara et al., 2014). However, there were some core genes that were not conserved at the amino acid level between the two geographic ChHV5 strains, including F-UL17 and F-UL15B, which are both involved with DNA processing and packaging (Table 3, *Ka*). The most divergent proteins at the amino acid level included the Ser/Arg-rich protein F-US2 and the hypothetical proteins HP17 and HP38 (Table 3, *Ka*).

Glycoproteins coat virions and play a major role in viral spread because of their critical role in virus-cell adhesion and recognition (Szpara et al., 2011). Functional constraints on glycoproteins vary, as glycoproteins were among both the most conserved and divergent genes among strains of HHV-1 (Szpara et al., 2014). Similarly, in six of the seven known glycoproteins we sequenced for both geographic ChHV5 strains, a few were among the most conserved genes sequenced (glycoproteins L and M), while others were less conserved at the amino acid level, with a maximum divergence of 1.12% (glycoprotein D, F-US4; Table 3, *Ka*), which is approximately half the maximum divergence estimate for HHV-1 glycoproteins (2.3%, Szpara et al., 2014).

Most of the ORFs, including the glycoproteins and many of the genes with higher levels of inter-region divergence mentioned above, had *ω* values less than one, indicating the predominance of synonymous substitutions and purifying selection (Table 3, *ω*). However, several proteins had *ω* estimates greater than one (F-UL03, F-lec2, plus the hypothetical proteins HP14, HP22, and HP38; Table 3, *ω*), which may be indicative of positive selection. For most of these genes, the overall level of divergence was low (*Dxy* ≤1%, Table 3), making *ω* challenging to estimate. However, divergence at HP38 (ICP4b) was among the highest observed (4.8%, Table 3, *Dxy*), and both synonymous and nonsynonymous substitutions were more numerous than in other genes with *ω* values greater than one.

Interestingly, the ChHV5 genome (Ackermann et al., 2012) contained four genes that are atypical for alphaherpesvirus genomes: two C-type lectin-like domain superfamily (F-lec1, F-lec2), a viral sialtransferase (F-sial), and an orthologue to the mouse cytomegalovirus gene M04 (F-M04). These genes were hypothesized to play a role in pathogenesis or immunodeviation and steps were taken in that study to verify their proper annotation. It is therefore worth noting that sequences for three of the four ORFs (F-lec1, F-lec2, F-sial) were recovered in this study and they revealed levels of evolutionary conservation comparable to other ChHV5 ORFs. This provides strong confirmation that these ‘atypical’ genes are not artifacts or insertions in a single lineage, but conserved and functional components of the ChHV5 genetic repertoire. However, the F-lec2 gene had an *ω* of slightly greater than one (1.064, Table 3), suggesting more relaxed evolutionary constraints relative to most of the examined ORFs.

Our sequencing produced full length sequence reads for 22 of the 38 potential coding sequences designated as hypothetical proteins by Ackermann et al. (2012). The lack of disruptive nucleotide changes and evolutionary rates comparable to ORFs of known function support the coding potential for the majority of these HPs. However, stop codons were detected for all FL variants but none of the HI variants within the coding regions for three HPs: HP25 (amino acid 151); HP31 (amino acid 150), and HP38 (amino acid 174).

We used the Shimodaira–Hasegawa (SH) test (Shimodaira & Hasagawa, 1999) implemented in PAML4.2 to test the concordance of phylogenies estimated for each ORF with a global topology determined below. This provides a statistical basis for identifying loci that exhibit novel phylogenetic patterns, without a presumption of the underlying cause (such as recombination, gene conversion, or major changes in evolutionary rate). Only ORFs that contained overlapping sequence data for all eight variants, i.e., ORFs for which a full locus-specific tree could be compared to the whole-genome phylogeny, were used. Out of 27 SH tests, three adjacent ORFs (F-UL28, F-UL29 and F-UL30) individually approached significance. Given the low number of variable sites used to perform this test, its discriminatory power is likely also low. The genomic clustering of these discordant ORFs and the fact that the nature of the discordance was the same in each case, i.e., that sample FL_USF clustered with HI samples (Fig. S1), adds weight to the biological significance.

### Genome-scale phylogeny compared to existing phylogeographic structure

We performed a genome-scale phylogenetic analysis to investigate possible changes between older and more recent HI samples and to generate a single best estimate of topology and branch lengths for evolutionary analysis of individual ORFs. Regions suspected to be recombinant based on the evidence described below, plus those with little sequence data, were masked (see Table 3; Masked).

**Figure 3:**
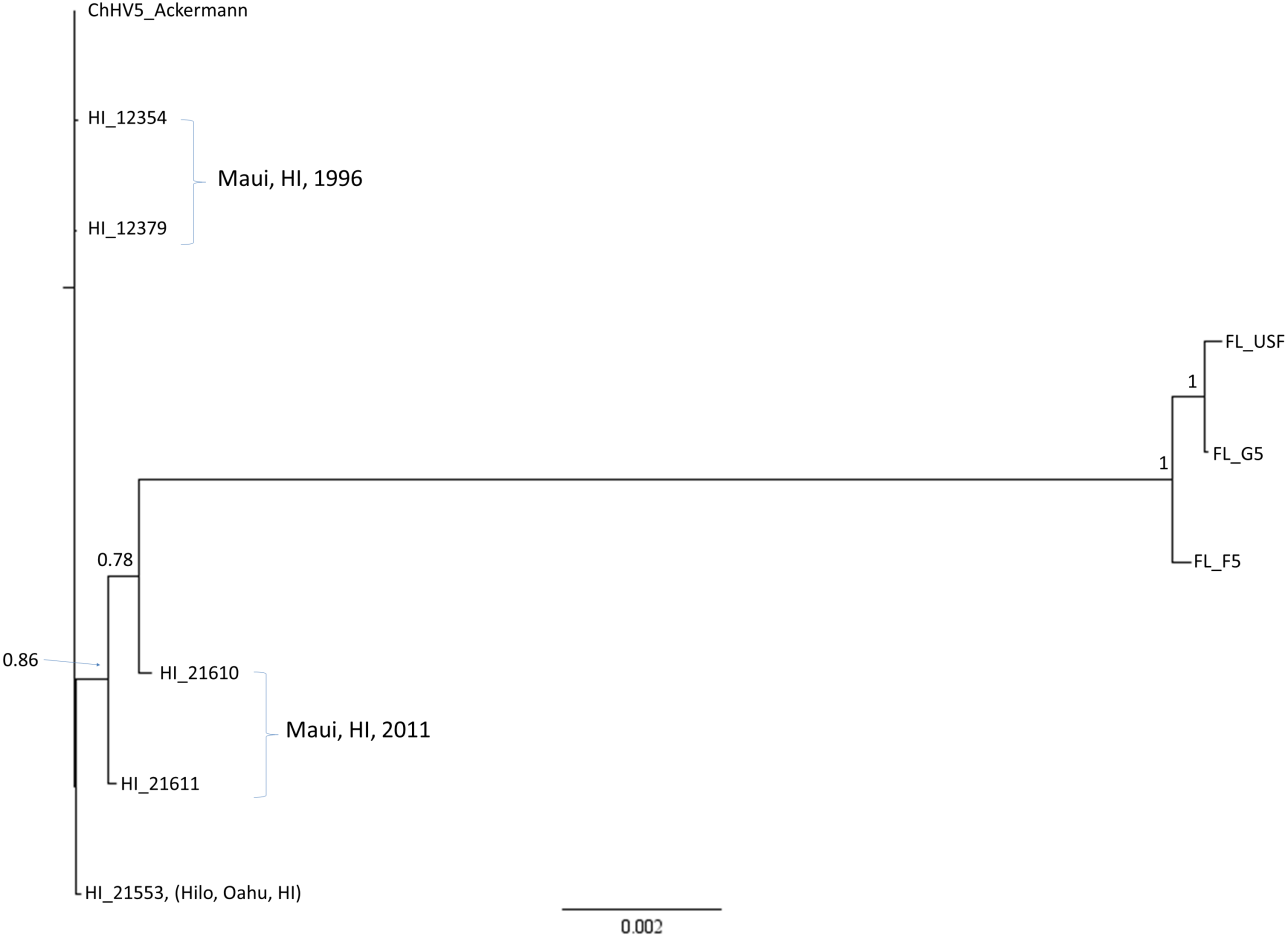
Global phylogenic inference based upon a concatenated alignment of 55 ChHV5 protein-coding genes (72,828 bp) for six Hawaii and three Florida strains. Shown is a mid-point rooted consensus tree based upon a Bayesian analysis with posterior probability support values above nodes.

Sequences for eight ChHV5 variants plus the reference genomic sequences covering 55 complete protein-coding genes were concatenated into an alignment that was 72,828 bp in length. Bayesian phylogenetic analysis produced a well-supported topology in which each variant was unique (Fig. 3). The FL variants formed a well-supported clade containing two similar variants (FL_F5 and FL_G5), while the third variant (FL_USF) was more differentiated. One Hawaiian variant sampled in Maui in 2011 (HI_21610) was slightly differentiated from the other Hawaiian variants, which were minimally differentiated from each other.

**Figure 4:**
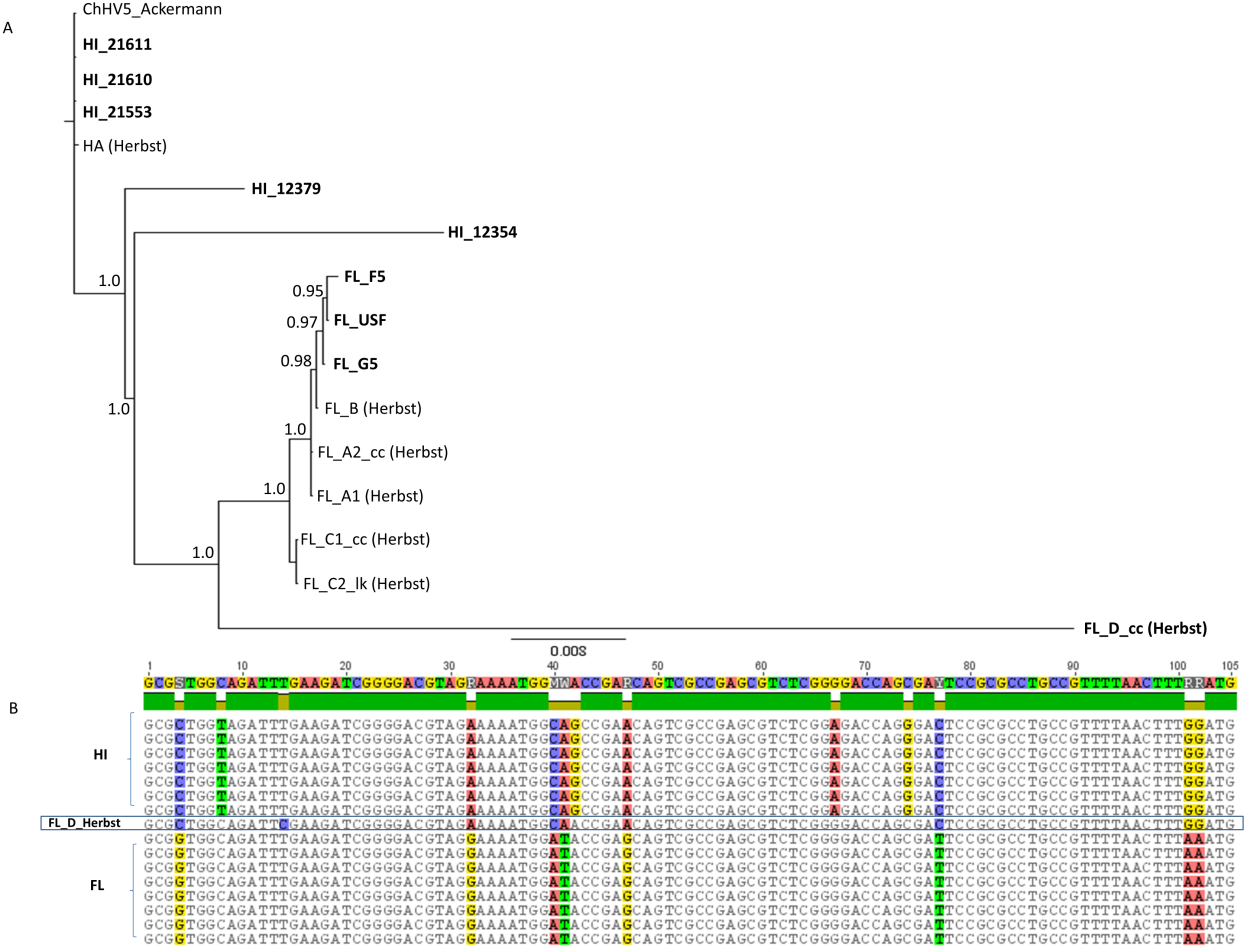
Phylogenetic inference based upon a 6,280 bp concatenated alignment of four gene regions following Herbst et al. (2004): Amplicons IV and V, DNA polymerase, and glycoprotein B. (A) Shown is a mid-point rooted consensus tree based upon a Bayesian analysis with posterior probability support values above nodes. Bolded names are sequences unique to this study. (B) Portion of the Amplicon V alignment showing the recombinant background of the FL_D sample, with eight of 12 fixed differences matching the Hawaii substitutional pattern.

We analyzed these newly sequenced FL and HI ChHV5 variants in the phylogeographic context of those identified previously by Herbst et al. (2004). A concatenated 6,280 bp alignment was constructed from partial F-UL30 (DNA polymerase; 2,019 bp), partial F-UL27 (glycoprotein B; 1,691 bp), plus the Amplicon IV (1,214 bp) and Amplicon V (1,356) ChHV5 genomic regions examined in Herbst et al. (2004). A Bayesian phylogenetic analysis of the alignment grouped HI and FL variants together (Fig. 4). Two of the newly sequenced Hawaiian variants, HI_21611 and HI_21553, were identical to the HA variant of Herbst et al. (2004) in this genome region. Similar to the whole genome phylogeny, HI_12379 and HI_12354 were slightly differentiated from the Hawaiian variants. The newly sequenced Floridian variants were closely related to the FL_B variant of Herbst et al. (2004), yet were not identical, differing at 1-5 bases. The FL_G5 variant was intermediate between the Herbst et al., FL_B variant and the other two newly sequenced FL variants, FL_USF and FL_F5. The FL_D variant (Herbst et al., 2004), that originated from a loggerhead sea turtle, was intermediate between the Hawaiian and Floridian variants and was basal to the clade containing all FL variants, yet was quite divergent (note long branch length leading to FL_D). There were 98 fixed differences between the regional clades, and the FL_D sequence matched the Hawaiian variants instead of the Floridian variants at 48 sites (see partial alignment, Fig. 4B).

### Recombination is a common feature of ChHV5 evolution

**Figure 5:**
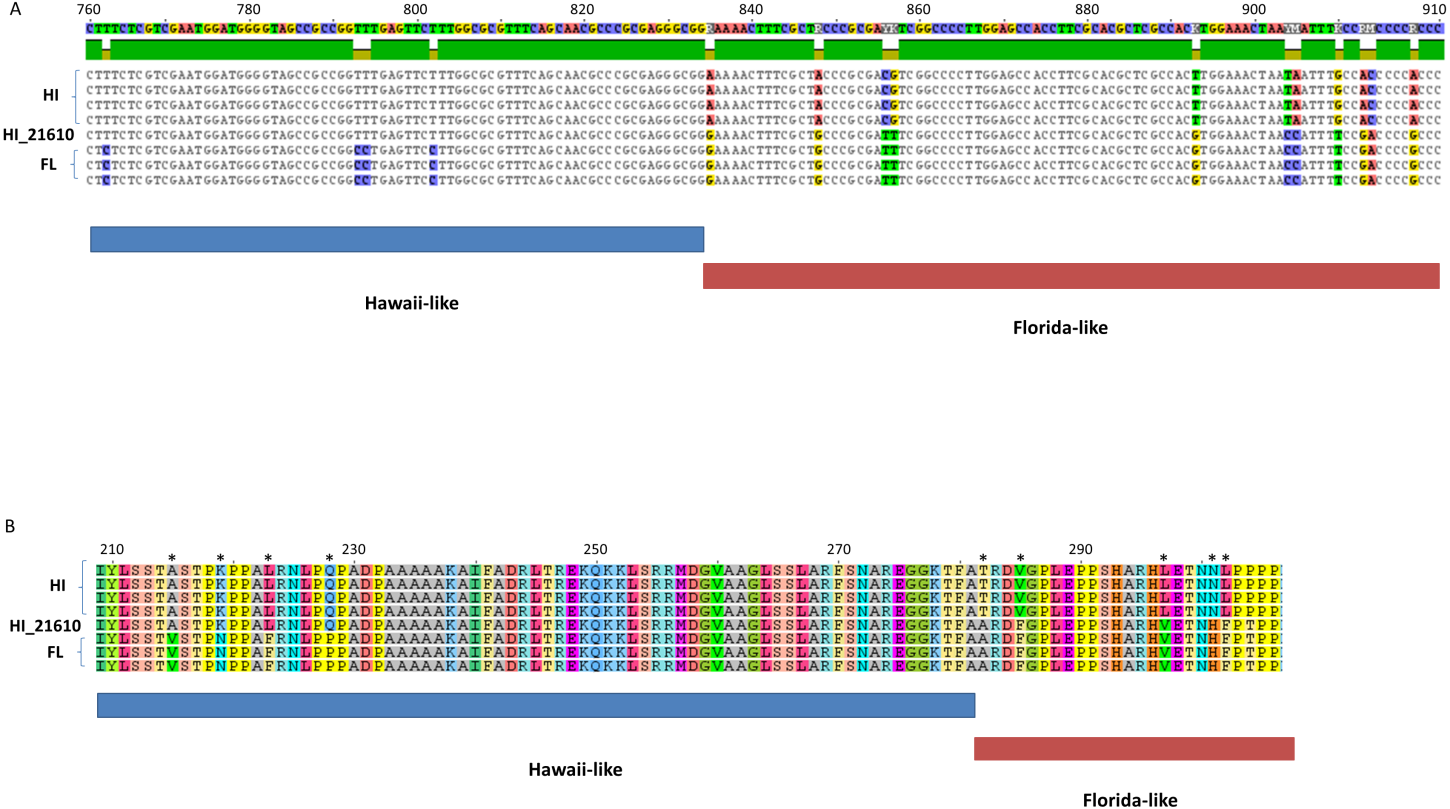
Partial DNA sequence (A) and amino acid alignments (B) for the F-UL17 gene. Note the transition of the HI_21610 sample from Hawaii-like (indicated by blue bar below alignments) to Florida-like (indicated by red bar below alignments, with variable alignment positions indicated by breaks in the green bar above the alignment in (A) and with * in (B).

Several lines of evidence reveal recombination to be an important mode of ChHV5 evolution, as in other alphaherpesviruses (Thiry et al., 2005; Szpara et al., 2014). First, unambiguous examples of recombination between FL and HI strains were identified. Analysis of ORF-specific phylogenies showed discordant placement of HI_21610 for ORFs in a window between approximately 50–55 kb of the reference genome. Visual inspection of the genome alignment in this region revealed many shared polymorphisms between HI_21610 and FL variants. For example, in the DNA sequence alignment for packing tegument protein F-UL17 variants, the HI_21610 variant matched the Hawaiian regional pattern of substitutions (fixed differences) for a portion of the alignment (bases 1–830), then matched the Floridian regional pattern for the second portion of the gene (Fig. 5A). The shared substitutions between HI_21610 and FL strains were also apparent in the amino acid alignment, in which 10 of the 27 variable amino acids matched FL variants for HI-21610 (Fig. 5B). The tests for recombination of the HI_21610 variant in this genomic region (50,146–68,418 bp) were highly significant (Table 4).

**Table 4:** *P*-values for the null hypothesis of no recombination between Florida and Hawaii ChHV5 strains. Five genomic regions (rows) were tested with six statistical tests of recombination (columns), as implemented in RDP4 (Martin et al., 2015).

	Breakpoints		Detection Methods					
Test Region	Begin	End	RDP	GENECONV	Bootscan	Maxchi	Chimaera	3Seq
FL_F5: 8762-21206	14,573	15,285	9.32E−11	2.69E−09	9.40E−11	7.42E−09	1.59E−08	6.44E−10
FLUSF: 42347–47743	44,848	45,560	1.22E−09	4.66E−08	1.16E−09	8.03E−05	7.53E−05	6.43E−07
HI21610: 50,146–68,418	51,900	54,755	7.69E−51	5.39E−47	5.97E−35	4.44E−23	4.31E−06	1.11E−16
FLD: 57235–58590	57,774	58,574	9.72E−04	3.68E−04	1.78E−04	4.49E−08	8.54E−04	5.06E−05
HI12534:103832–104365	104,148	104,271	NS	0.0459	NS	NS	NS	NS

**Notes.**

a Breakpoints were identified by RDP4 and are represented by alignment coordinates in bp.

Additional lines of evidence suggest that recombination with unsampled variants has occurred. The first is the distribution of Tajima’s *D* calculated for ORFs along the genome, revealing genomic regions with either positive or negative values of this statistic. Strongly negative *D* values indicate an excess of low-frequency polymorphisms. Indeed, examination of alignments in regions with negative Tajima’s *D* frequently coincided with stretches of private alleles not found in any other sample (Fig. S2). We therefore plotted the number of private alleles for each sample in 100-bp windows along the genome, transformed as a Z-score in which the mean and standard deviation are from all such windows across all sequenced samples. Values are color-coded to produce a heatmap in which the highest rates of private alleles are red and the lowest values are dark gray (Fig. S3). Several long tracts of private alleles were evident, for example, from positions 14,000–21,000 for sample FL_USF. This region corresponds to several genes in the US region of the genome (F-US1, F-US2, and F-US3A and B, F-US4) that also had high proportions of segregating sites and negative Tajima’s *D* values (Table 3). Another concentration of singletons for the FL_USF variant occurred in the F-UL07 through F-UL09 ORFs, and two significant break points were detected (FL_USF: 42347–47743; Table 2). The HI_12354 variant had many singletons in the F-UL28 through F-UL30, plus the F-UL38 through F-UL52 ORFs.

## Discussion

High throughput sequencing strategies and large nucleotide dataset additions to public databases now provide a means to augment our understanding of herpesvirus evolution (McGeoch, Rixon & Davison, 2006; Abdelgawad et al., 2016). Genome resequencing of spatially segregated ChHV5 variants provides an in-depth examination of strain variation at the DNA and amino acid levels to facilitate comprehensive phylogeographic analyses that were previously not feasible. Through a combination of long-range PCR amplification and amplicon sequencing adaptable to any high-throughput platform, we have developed an approach to successfully sequence the majority of ChHV5 protein coding genes from unenriched host extracts. With current Illumina HiSeq output, for example, hundreds of samples could be multiplexed together, although an optimized pooling strategy that accounts for variable efficiency of long-range PCRs by region merits further exploration. Similar custom strategies are frequently utilized for virus resequencing (Kvisgaard et al., 2013; Tweedy et al., 2016). This method is cost-effective for the examination of genomic evolution of ChHV5 across space and time, and facilitates the identification of both quickly evolving genes that may be useful for molecular epizootiological studies, and more slowly evolving genes that may be of fundamental importance to viral infection. Since there is no clear, practical understanding of how genetic differences between alphaherpesviruses influence pathogenesis, the detailed examination of viral genomes is likely to lead to an increased understanding of the origins, pervasiveness, and flexibility of viral functions, and the factors that have shaped viral evolution through time.

This study documents divergence between Western Atlantic and Eastern Pacific ChHV5 strains from green sea turtles (*C. mydas*) within a majority of the protein coding genes of ChHV5. Many of these genes are examined for geographic variability for the first time, including seven genes in the US region and 46 genes in the UL region. Similar to previous studies, the majority of genomic variation was observed between geographic locations, with a total of 1,001 fixed differences detected between FL and HI variants. As suggested by previous molecular studies (Quackenbush et al., 1998; Quackenbush et al., 2001, Greenblatt et al., 2005a; Greenblatt et al., 2005b; Duarte et al., 2012; Patrício et al., 2012; Rodenbusch et al., 2012; Monezi et al., 2016), the divergent strains match predominantly regional movement patterns of sea turtle hosts, indicating that FP is geographically specific (Herbst et al., 2004; Ene et al., 2005; Patrício et al., 2012; Jones et al., 2016; Ariel et al., 2017). Variability in rates of sequence divergence among ChHV5 genes was consistent with results noted for other alphaherpesviruses such as HHV-1, where the most divergent proteins were approximately 2.8% divergent (Szpara et al., 2014). However, it is now possible to examine these genes in ChHV5 at a fine scale to better understand how encoded proteins are evolving and what evolutionary forces may shape geographic strain divergence.

The Amplicon IV and V regions of ChHV5 (Herbst et al., 2004) are commonly utilized for phylogenetic analyses. Here we documented that these target regions contain some of the most variable genes (HP17 and F-UL18, respectively), and quantitatively validated their utility for the determination of geographic variants. Interestingly, some of the variability observed in these gene regions was likely introduced through recombination (see below). We also detected several ORFs with *ω* values greater than one (F-UL03, Flec2, and HP14, 22 and 38) that were accumulating coding substitutions faster than expected under strict neutrality, which may suggest loss of functional constraint. The UL03 gene was found to be conserved in a study of HHV-1 (Karamitros et al., 2016). Divergence estimates for the other four genes (Flec2, and HP14, 22 and 38) are not comparable with studies of other herpesviruses, as these are specific to ChHV5. We did not perform a secondary likelihood ratio test to see if these estimates were significantly greater than one, as we believe the small number of samples lacks statistical power to robustly implement that test. Rather, these ORFs merit further analysis with targeted sequencing in a larger set of samples.

Overall, variability within geographic locations was low, yet in each location, we detected more variants than have been documented previously. Notably, the three samples from FL sequenced for this study were each unique and although all three were most closely related to the FL-B variant, which is common in the Indian River Lagoon (Ene et al., 2005), none matched the four variants (FL A-D) previously documented from FL coastal waters (Herbst et al., 2004; Ene et al., 2005). The approximately 6-kb fragment initially used by Herbst et al. (2004) to determine virus variants does not capture the divergence that one of our newly sequenced variants (FL_USF) exhibits when additional protein coding genes are examined, suggesting that previous studies may have underestimated strain diversity. Given that several genes in the US genomic region distinguished the FL_USF variant, inclusion of sequence data from these genes may better distinguish FL variants. Similarly, each of the samples sequenced from HI was unique, yet genetic differences among these variants were fewer than those among FL variants.

Homologous recombination, the replacement of a genomic region with sequence from a co-infecting genome via homology-mediated strand exchange, is a feature known to influence alphaherpesvirus evolution (Davison, 2000; Thiry et al., 2005; Thiry et al., 2006). Specifically, other alphaherpesviruses, such as HHV-1 (Bowden et al., 2004), bovine herpes virus 1 (BoHV-1; Thiry et al., 2006), equine herpesviruses (EHV-1 EHV-4 and EHV-9; Pagamjav et al., 2005; Abdelgawad et al., 2016), and varicella-zoster virus (Norberg et al., 2015) undergo recombination. The establishment of a latent viral infection may increase the likelihood for co-infection and in turn, recombination (Ma, Azab & Osterrieder, 2013). As such, recombination may be a driving evolutionary force that introduces variation against a background of more conservative evolution known in alphaherpesviruses (Thiry et al., 2005).

The genomic sequencing of geographic strains of ChHV5 from FL and HI green sea turtles presented here has provided the first compelling evidence of recombination among strains of ChHV5; a previously undescribed yet likely important aspect of ChHV5 epizootiology. Based upon visual inspection of sequence alignments, there was clear evidence of recombination between FL and HI geographic strains in a few ORFs (F-UL12 and F-UL17; see Fig. 5B). These examples are particularly clear because multiple sequences from both regions are available and the two regional strains are strongly diverged. Additionally, for several of the variants sequenced for this study, clusters of singleton substitutions were detected that could be more parsimoniously explained by recombination with an unsampled lineage rather than many individual mutations. Genomic regions that contained potential recombinant sequences were suggested by a phylogenetic pattern that did not match the overall topology, strongly bimodal Tajima’s *D* statistics, and highly clustered distributions of unique alleles. Several specific instances of hypothesized recombination between FL and HI types were subsequently confirmed by highly significant *p*-values for a suite of recombination tests. Although lineage-specific diversifying selection or lineage-specific mutational hotspots remain formal possibilities, the recognition of recombination as a driving force in the evolution of other alphaherpesviruses, plus the data presented here, point to recombination as the likely cause of high rates of polymorphism observed in certain regions of the genome. It is important to note that clusters of private alleles are not limited to coding regions or enriched for nonsynonymous changes, discounting selection on protein sequence as a possible cause. There is also no obvious indication of mutational hotspots, as there is no tendency for clusters of private alleles to occur in the same region in different samples. The only reasonable explanation we can postulate for the patterns in Fig. S3 is recombination between the viral lineage and another divergent lineage not represented in our sample.

The complex life histories of sea turtles are likely to have important implications to the regional distribution of ChHV5 strains (Patrício et al., 2012). After spending the first few years in oceanic environments, juveniles form aggregations of individuals from multiple nesting colonies and even different species in coastal foraging habitats (Bowen & Karl, 2007). Adults often undergo long-distance migrations (hundreds to thousands of kilometers) to mating areas and nesting beaches (Bowen & Karl, 2007). Phylogeographic analyses involving green sea turtles have confirmed that there is sufficient exchange between ocean basins to prevent long-term isolation and allopatric speciation (Roberts, Schwartz & Karl, 2004; Bourjea et al., 2007). Leatherbacks are tolerant of cold waters and have a distribution spanning from the tropics to the Arctic Circle, showing no evolutionary partitions between the Indo-West Pacific and the Atlantic and no geographic segregation of lineages by ocean basin (Bowen & Karl, 2007). Other more temperate species, such as loggerheads, ridleys and hawksbills are known to congregate at feeding grounds, and it has been noted that the swarm of these species in Brazil may create an important phenomenon for the evolution of sea turtles (Bowen & Karl, 2007). Such overlapping distributions of sea turtle hosts may create opportunity for inter-species transmission and for co-infection by divergent virus strains that are dominant in different sea turtle species. Although interspecies transmission is unusual for most alphaherpesviruses (Davison, 2000), it has been documented for ChHV5 in regions where multiple sea turtle species co-occur (Herbst et al., 2004; Ene et al., 2005; Patrício et al., 2012). In such scenarios, recombination between divergent strains may give rise to novel variants that may differ in virulence. In this way, even slowly evolving viruses, such as ChHV5 (Herbst et al., 2004; this study), may diversify rapidly.

In light of this information, it is interesting to note that in several phylogeographic analyses of ChHV5, highly divergent strains have been documented from FL waters, such as the FL_D haplotype (Herbst et al., 2004; Ene et al., 2005; called FL_3 variant by Patrício et al., 2012). Inconsistent phylogenetic patterns led to the detection of recombination in the FL_D variant, yet the results were discounted as potential artifacts resulting from mis-identifications in GenBank sequences (Patrício et al., 2012). It has also been hypothesized that highly divergent variants such as FL_D were restricted to different species of sea turtles including the Atlantic loggerhead (Herbst et al., 2004). Yet divergent lineages have been reported in Pacific olive ridley turtles, and in a green sea turtle from San Diego (Patrício et al., 2012). Visual inspection of the sequence alignment of the 6,160 bp ChHV5 segment utilized by Herbst et al. (2004) (Fig. 5A) shows that the FL_D variant shares many fixed substitutions with HI strains as well as FL strains (Fig. 5B), suggesting this lineage may be the result of a previous recombination event that involved divergent oceanic strains. As suggested by Jones et al. (2016), our results suggest that further genomic profiling of ChHV5 from different regions is essential for improving our understanding of zootiology and pathogenesis of this virus.

Now that recombination has been clearly demonstrated among ChHV5 strains, future work should examine whether certain genes are more likely to have histories including recombination. For example, a high frequency of substitutions was observed in HP38 (ICP4b) in our dataset, which is consistent with known recombination in the ICP4 gene of equid herpesviruses (Pagamjav et al., 2005). Also, the genomic feature of inverted repeats shared by the subfamily *Alphaherpesvirinae* allows for segment inversion due to recombination during DNA replication, given adequate sequence similarity (Davison, 2000; Thiry et al., 2005). Efforts to amplify the two approximately 8-kb inverted repeat regions between the US and UL were not successful, in line with previous reports that these regions are recalcitrant to standard PCR protocols given low conservation rates, presence of microsatellite loci, and palindrome stem loops (Zhao, Haqqi & Yadav, 2000; Kieleczawa, 2005; Kieleczawa, 2006; Karamitros et al., 2016). In HHV-1, the inverted repeat regions plus a 400-bp terminal redundancy play a key role in recombination between the long and short genomic segments (McGeoch et al., 1986; McGeoch et al., 1988). These recombination events are thought to be essential for viral replication and *in vivo* infection (Jenkins & Roizman, 1986; Jenkins & Martin, 1990). Given this importance of the inverted repeat region in other herpesviruses, future studies could strive to recover full genomic sequences from different isolates. Typically, repeat regions in herpesviruses are determined by cloning of purified viral DNA from monolayer cell cultures into plasmids (McGeoch et al., 1991), an approach not currently feasible with ChHV5 that can only be grown with difficulty in organotypic cultures (Work et al., 2017). The addition of long-read high throughput sequencing may prove useful (e.g., Karamitros et al., 2016).

Methodologies that estimate the prevalence of ChHV5 co-infection, a precursor to recombination, as well as those that determine the relationship between virus genotype and disease phenotype (comparative pathology), should be priority topics of future research. The difficulty in isolating and culturing ChHV5 in the laboratory hampers more traditional approaches to investigate recombination rates by capturing concatemers (e.g., Slobedman, Zhang & Simmons, 1999; Meurens et al., 2004) and by assessment of the biological properties of recombinant strains (e.g., Muir, Nichols & Breuer, 2002). However, the next-generation sequencing approach described here should prove useful in this endeavor, as the sequencing of numerous global variants from all sea turtle species will allow for more robust statistical tests for recombination and better characterization of the frequency and implications of recombination events. Through additional ChHV5 genomic sequencing, researchers have access to a greater array of diagnostics. A coordinated effort will allow integration of datasets, including population genetics of the virus and host species, in addition to molecular and epizootiological views of regional and global infection, for a comprehensive understanding of ChHV5 biology, virulence and transmission, potentially leading to more accurate risk assessments and disease mitigation strategies.

## Conclusions

The genome re-sequencing of spatially segregated ChHV5 variants presented here allows for an in-depth examination of strain diversity at the nucleotide and amino acid levels. Genome-wide SNPs confirm broad divergence of geographic ChHV5 strains and general conservation at the protein level. However, this resequencing effort has provided the first compelling evidence that recombination is a common feature of ChHV5 evolution, and is likely an important aspect of the epizootiology of fibropapillomatosis. In the future, we suggest surveys of turtles in coastal foraging habitats, where an overlapping distribution of sea turtle species may provide the opportunity for inter-species ChHV5 transmission, co-infection by divergent virus strains, and possibly novel variants that may differ in virulence.

##  Supplemental Information

10.7717/peerj.4386/supp-1 Table S1 Long-range PCR primers used to amplify ChHV5 genomic sections in green sea turtles

10.7717/peerj.4386/supp-2 Table S2 High throughput sequencing runs, reads, and mapping success to the ChHV5 genome

10.7717/peerj.4386/supp-3 Figure S1 Alternative placement of samples HI_12354 and HI_12379 for three ChHV5 genes with SH tests that approached significance (A) Topology of the global phylogeny (B) Phylogenies for F-UL28, 29 and 30, showing that the different placement of the HI-12354 and HI_12379 samples.

10.7717/peerj.4386/supp-4 Figure S2 Estimates of Tajima’s *D* statistic (Tajima, 1989) for ChHV5 ORFs (A) Plotted as a histogram (B) Ordered by genomic position.

10.7717/peerj.4386/supp-5 Figure S3 Heatmap of singleton substitutions along the length of the ChHV5 genome, calculated in 500 bp sliding windows

## Additional Information and Declarations

### DNA Deposition

The following information was supplied regarding the deposition of DNA sequences: Raw sequence data has been submitted to the NCBI short read archive (SRA), bioproject # PRJNA360405, including biosample numbers SAMN06210144, SAMN06210145, SAMN06210146, SAMN06210147, SAMN06210148, SAMN06210149, SAMN06210150.

### Data Availability

The following information was supplied regarding data availability: Metadata, as well as processed alignment files associated with this project can be found at USGS ScienceBase, Morrison, C.L., Cornman, R.S., Iwanowicz, Luke, Iwanowicz, Deborah, Adams, C.R., and Sanders, L.R., 2018, Targeted amplicon resequencing of chelonid alphaherpesvirus 5 from tumor samples collected from *Chelonia mydas* (green turtle) inhabiting Hawaiian and Florida coastal waters: US Geological Survey data release, 10.5066/F73B5Z3T.
