# Supplementary material for: Genomic evolution, recombination, and inter-strain diversity of chelonid alphaherpesvirus 5 from Florida and Hawaii green sea turtles with fibropapillomatosis
Source: PeerJ. 2018 Feb 20;6:e4386. doi: 10.7717/peerj.4386 (PMC5824677; doi:10.7717/peerj.4386)
Supplement: Table S1 [file peerj-06-4386-s001.docx]

| Primer set | Forward primer | Primer sequence | Reverse Primer | Primer Sequence | Product  size | HI | FL |
| --- | --- | --- | --- | --- | --- | --- | --- |
| ChHV5-LSC-02 | 4110F | TGATGAAGAACCCGAACCCG | 9076R | TGACGGGAAAAGAGGGCTTC | 4966 | Y | N |
| ChHV5-LSC-03 | 7756F | AAAAGGCTTTCTCGGAGGGG | 13013R | GCTGCAAATCTCTGCTCGTG | 5257 | Y | N |
| ChHV5-LSC-04 | 12112F | TCGCGTTATCTGACGAGCTC | 16987R | GAGATCCTTACTGCCGGACG | 4875 | Y | Y |
| ChHV5-LSC-05 | 15665F | AGTAGAGGTTCGGCTTTGGC | 21211R | CGGACAGCATTTCATCTGCG | 5546 | Y | Y |
| ChHV5-LSC-06 | 20658F | CGTGCTATTTGAATGCCCCG | 26071R | ATCATCGCCACCGGATGTAC | 5413 | Y | Y |
| ChHV5-LSC-09 | 34335F | CCATAATCGGACGGTCCTGG | 39730R | TGCCTGTGTTTCCTCTCGTC | 5395 | Y | N |
| ChHV5-LSC-10c | 38150F | GCTCATCACCGAGACGAAAC | 46093R | GCTTTTTCAACGCGGTCATG | 7943 | Y | N |
| ChHV5-LSC-10b | 38840F | CATCAGAGCCCTTTCGAAGC | 46766R | AGTTTTGACGCCGAGCATTT | 7926 | Y | N |
| ChHV5-LSC-10a | 39688F | TCTTGATCAGGTTGGGCGTC | 45529R | CCAAAGGGGTGGATGTCTCC | 5841 | Y | Y |
| ChHV-USF-2A | 39697F | ATCAGGTTGGGCGTCCCCGA | 45445R | TACAGGCAGCGAGGTTTTTC | 5748 | Y | Y |
| ChHV5-LSC-11 | 45288F | TTTACGGAGCACACCTCACC | 50531R | GGAGACGATCCTTCAGCGAG | 5243 | Y | Y |
| ChHV5-LSC-12 | 50001F | AAGTCCGCGAACGTTTTTGG | 55036R | AAAGACCCGAAGCCTTGAGG | 5035 | N | Y |
| ChHV5-LSC-13 | 54762F | ACGCGCCAAAGAACTCAAAC | 59099R | AGCTCTGTCAGAAACCGAGC | 4337 | Y | Y |
| ChHV5-LSC-13b | 54796F | CTACCCCATCCATTCGACGA | 59702R | CGGCCGCCAAGGTTTTTATT | 4862 | Y | Y |
| ChHV5-USF-3A | 54804F | ACCCCATCCATTCGACGAGA | 59657R | ACGGAGCGCAATGTAGAGTT | 4853 | Y | Y |
| ChHV5-LSC-14 | 58504F | TGGGCTCGTAAACTTCGGAC | 63624R | CGGCGAAAGATAACCGCTTG | 5064 | Y | Y |
| ChHV5-LSC-15 | 62964F | GAAGTCTCCGCGTACCTAGC | 68428R | CGATTCGGCTCTTTCGCAAG | 5464 | Y | Y |
| ChHV5-LSC-16 | 67715F | TTTTCGACAAGCACGGCATG | 72955R | GCGATTGATCCAACGTGAGC | 5240 | N | Y |
| ChHV5-LSC-17 | 72610F | TTCCTCTTCGGGGGAGAGAG | 77568R | ATGAACGAAGACCTGCTGGG | 4958 | Y | Y |
| ChHV5-LSC-18 | 73394F | TCTGCCACAGCTTCAGAGTG | 82866R | AACGCCAAGACTGAAGAGGG | 5472 | Y | Y |
| ChHV5-LSC-19 | 82303F | AGACCGGTAAAAGGCTTGGG | 87753R | GCGTACCGCGTGTTATTTCC | 5450 | Y | Y |
| ChHV5-LSC-20c | 85936F | ATCAAGGTTCGCATCAACGG | 92793R | ATCAGCCGATCGAAAACGTG | 6857 | Y | Y |
| ChHV5-LSC-20 | 87591F | CGGAACCCGCTCACAAAAAG | 92786R | GTCTTATTGGACGCGCGTTC | 5195 | Y | Y |
| ChHV5-LSC-21 | 91526F | AATCGTCGACCACACACCTC | 96800R | GGCGGTTTTATCGGTGATGC | 5274 | Y | Y |
| ChHV5-LSC-22 | 96260F | CCAATTCGATCGCGTTCCAC | 101633R | TTTTCAGCTGAAACGCCTGC | 5403 | Y | Y |
| ChHV5-LSC-23 | 101478F | TACATTCGACTCCGGCATCG | 106607R | ACGGGTGAGTCTTCGGTTTC | 5129 | Y | Y |
| ChHV5-LSC-24 | 106424F | CGTACGCCGTCAACGAAATC | 111576R | CGTGTTTTGCTTGGTGGGAG | 5152 | Y | Y |
| ChHV5-LSC-25 | 111236F | GTGGACGCAAAAGGGTTTCC | 116514R | TTTGCGAGCACAACTTGACG | 5278 | Y | Y |
| ChHV5-USF-5A | 111403F | CCTGACCAGACCGTGAATCT | 112772R | GCCACCGAATACCTTTTGAA | 1369 | Y | Y |
| ChHV5-LSC-26 | 11587F | TCGCTATCCGGTGCATAACC | 121021R | TTGCTTTTGGACAATGGCGG | 5144 | Y | Y |
| ChHV5-LSC-27 | 120901F | AAACGTGTTCCTTCGCTTGC | 125756R | TCCAGTCGAAACTCCGTTGG | 4855 | Y | Y |
| ChHV5-LSC-29b | 125269F | GCCTCCGATGAAAATGTCCC | 132124R | TGCCAGACTGCTTCTAACGG | 6855 | Y | N |
| ChHV5-LSC-28 | 125270F | TTTCACACCCTCTTCGAGCC | 130355R | GCAGATGTACGAGTACGGGG | 5085 | Y | N |
|  |  |  |  |  |  |  |  |
