# Supplementary material for: Genomic evolution, recombination, and inter-strain diversity of chelonid alphaherpesvirus 5 from Florida and Hawaii green sea turtles with fibropapillomatosis
Source: PeerJ. 2018 Feb 20;6:e4386. doi: 10.7717/peerj.4386 (PMC5824677; doi:10.7717/peerj.4386)
Supplement: Table S2 [file peerj-06-4386-s002.docx]

| Run ID | Sample ID | Read length | Total Reads | Mapped reads | % Mapped |
| --- | --- | --- | --- | --- | --- |
| AHCRE MiSeq | HI_21533 | 301 | 2,869,708 | 2,620,606 | 91 |
| AHCRE MiSeq | FL_F5 | 301 | 2,240,934 | 1,948,282 | 87 |
| AHCRE MiSeq | FL_G5 | 301 | 2,708,796 | 2,599,695 | 96 |
| AHCRE MiSeq | HI_12354 | 301 | 2,569,558 | 2,350,528 | 92 |
| AHCRE MiSeq | HI_12379 | 301 | 3,290,184 | 3,018,276 | 92 |
| AHCRE MiSeq | HI_21611 | 301 | 3,176,416 | 2,581,902 | 81 |
| AHCRE MiSeq | HI_21610 | 301 | 2,062,138 | 1,420,640 | 69 |
| AL1A4 MiSeq | FL_F5 | 301 | 11,377,404 | 10,917,297 | 96 |
| AL1A4 MiSeq | FL_USF | 301 | 11,647,216 | 731,328 | 6 |
| AL1A4 MiSeq | HI_12354 | 301 | 15,759,514 | 15,460,486 | 98 |
| AL1A4 MiSeq | HI_21610 | 301 | 8,073,662 | 7,897,682 | 98 |
| AFG3R MiSeq | HI_12354 | 300 | 14,602,156 | 11,969,741 | 82 |
| AFG3R MiSeq | HI_12379 | 300 | 4,251,536 | 3,802,003 | 89 |
| AFG3R MiSeq | HI_21533 | 300 | 9,765,490 | 7,105,215 | 73 |
| AFG3R MiSeq | FL_F5 | 300 | 4,876,420 | 3,924,303 | 81 |
| AFG3R MiSeq | FL_G5 | 300 | 4,426,734 | 3,973,699 | 90 |
| GPOQ9 Torrent | FL_F5 | 178 | 4,710,567 | 4,447,111 | 94 |
| RB0AC Torrent | HI_21533 | 116 | 2,107,387 | 1,747,934 | 83 |
| Average |  | 284 | 6,139,768 | 4,917,596 | 83 |
