# Supplementary figures and images for: Genomic evolution, recombination, and inter-strain diversity of chelonid alphaherpesvirus 5 from Florida and Hawaii green sea turtles with fibropapillomatosis
Source: PeerJ. 2018 Feb 20;6:e4386. doi: 10.7717/peerj.4386 (PMC5824677; doi:10.7717/peerj.4386)

# Global

A)

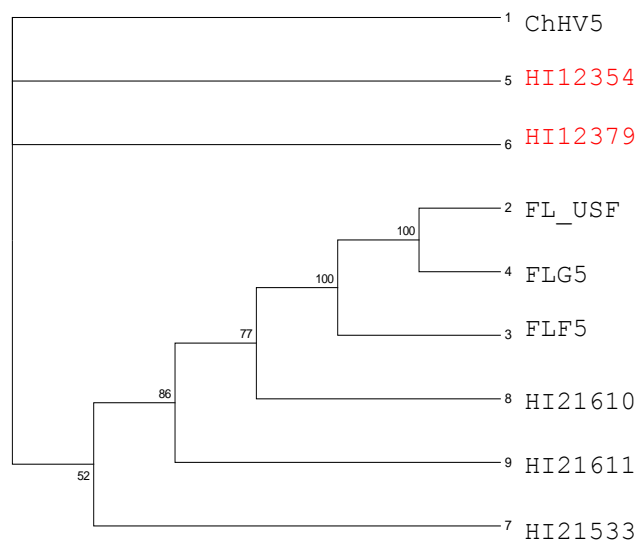

B) AHA93357, F-UL28

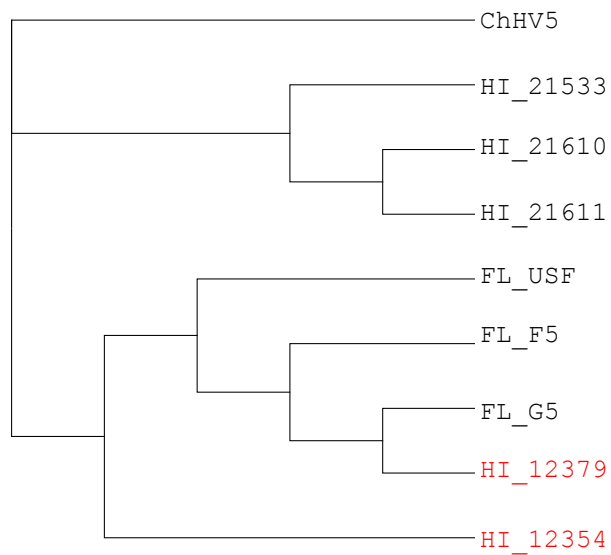

AHA93358, F-UL29

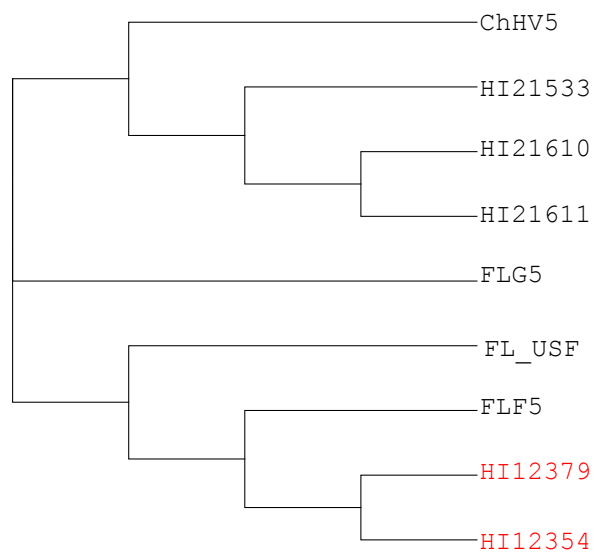

AHA93360, F-UL30

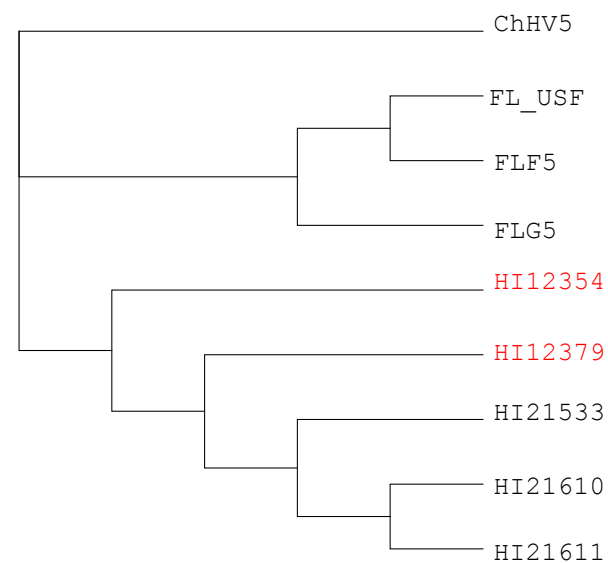

Supplement: Figure S1 — (A) Topology of the global phylogeny (B) Phylogenies for F-UL28, 29 and 30, showing that the different placement of the HI-12354 and HI_12379 samples. [file peerj-06-4386-s003.pdf]

A

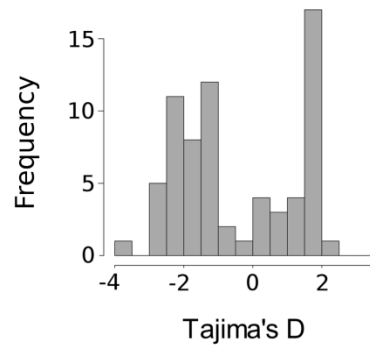

B

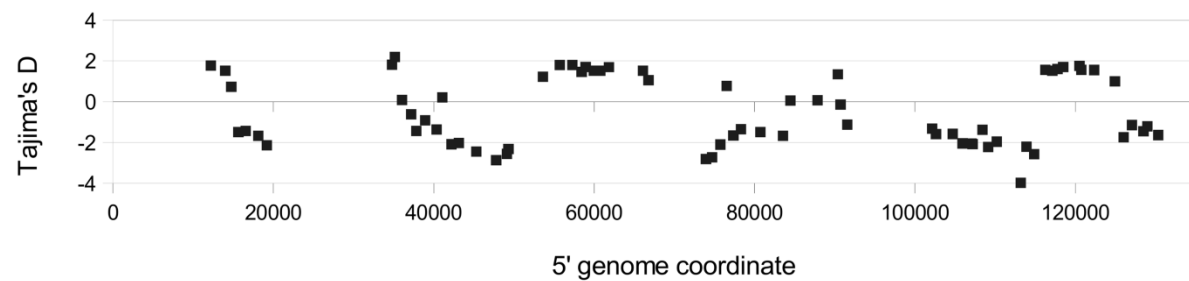

Supplement: Figure S2 — (A) Plotted as a histogram (B) Ordered by genomic position. [file peerj-06-4386-s004.pdf]

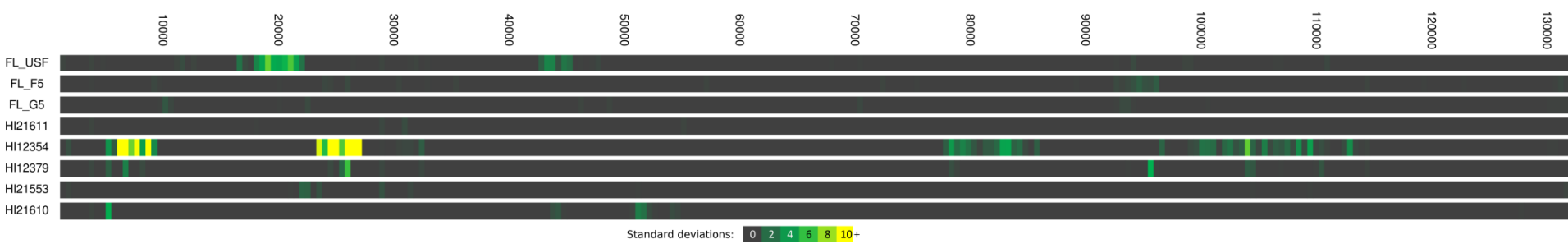

Supplement: Figure S3 [file peerj-06-4386-s005.pdf]
